# Overfishing of top predators eroded the resilience of the Black Sea system regardless of the climate and anthropogenic conditions

**DOI:** 10.1111/j.1365-2486.2010.02331.x

**Published:** 2011-03

**Authors:** Marcos Llope, Georgi M Daskalov, Tristan A Rouyer, Vesselina Mihneva, Kung-Sik Chan, Alexander N Grishin, Nils Stenseth

**Affiliations:** * Centre for Ecological and Evolutionary Synthesis (CEES), Department of Biology, University of OsloPO Box 1066 Blindern, NO-0316 Oslo, Norway; † CEFAS Lowestoft LaboratoryPakefield Road, Lowestoft, Suffolk NR33 0HT, UK; ‡ Centre de Recherche Halieutique Méditerranéenne et Tropicale, Institut Français de Recherche pour l'Exploitation de la Mer (IFREMER)Avenue Jean Monnet, BP 171, 34203 Sète cedex, France; § Institute of Fisheries and AquacultureVarna, PO Box 72, Varna 9000, Bulgaria; ¶ Department of Statistics and Actuarial Science, University of Iowa263 Schaeffer Hall, Iowa City, IA 52242, USA; ∥ Southern Scientific Research Institute of Marine Fisheries and Oceanography (YugNIRO)2, Sverdlov Street, 98300 Kerch, Crimea, Ukraine; ** Flødevigen Marine Research Station, Institute of Marine Research (IMR)NO-4817 His, Norway

**Keywords:** Black Sea, ecological thresholds, ecosystem resilience, eutrophication, GAM, regime shifts, scenarios, trophic regulation

## Abstract

It is well known that human activities, such as harvesting, have had major direct effects on marine ecosystems. However, it is far less acknowledged that human activities in the surroundings might have important effects on marine systems. There is growing evidence suggesting that major reorganization (i.e., a regime shift) is a common feature in the temporal evolution of a marine system. Here we show, and quantify, the interaction of human activities (nutrient upload) with a favourable climate (run-off) and its contribution to the eutrophication of the Black Sea in the 1980s. Based on virtual analysis of the bottom-up (eutrophication) vs. top-down (trophic cascades) effects, we found that an earlier onset of eutrophication could have counteracted the restructuring of the trophic regulation at the base of the food web that resulted from the depletion of top predators in the 1970s. These enhanced bottom-up effects would, however, not propagate upwards in the food web beyond the zooplankton level. Our simulations identified the removal of apex predators as a key element in terms of loss of resilience that inevitably leads to a reorganization. Once the food web has been truncated, the type and magnitude of interventions on the group replacing the apex predator as the new upper trophic level have no effect in preventing the trophic cascade. By characterizing the tipping point at which increased bottom-up forcing exactly counteracts the top-down cascading effects, our results emphasize the importance of a comprehensive analysis that take into account all structuring forces at play (including those beyond the marine system) at a given time.

## Introduction

**Fig. 1:**
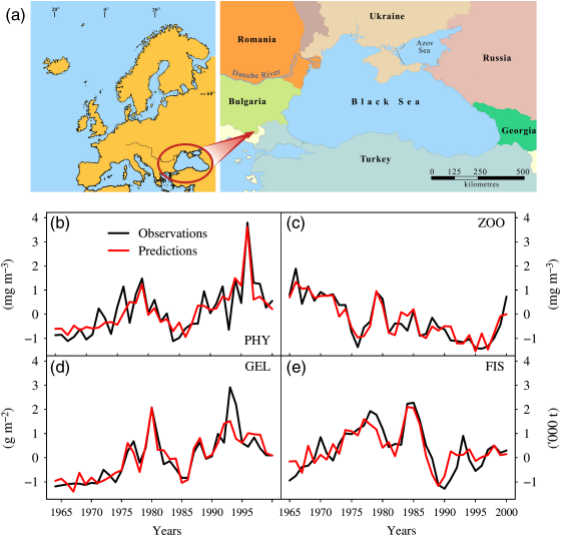
Black Sea and biological series. (a) Map showing the location of the Black Sea in Europe and the mouth of the Danube River. (b–e) Observations and predictions [as estimated from the individual generalized additive models, Eqns (1)–(4)] for phytoplankton, zooplankton, jellyfish, and planktivorous fish.

The Black Sea is a deep, mostly land-locked, basin in Eastern Europe. It is linked to the Mediterranean by the narrow straits of Bosporus and Dardanelles (Fig. 1a). The surrounding land area entertains intensive human activities and has experienced profound economical and societal changes in the formerly communist countries. That the Black Sea has undergone dramatic environmental changes in recent decades underlies its importance as a ‘natural laboratory’ for studying marine ecosystem dynamics (Mee *et al.*, 2005; Daskalov *et al.*, 2007; Oguz & Gilbert, 2007;).

The Black Sea is the world's largest meromictic basin consisting of a two-layer system separated by a permanent pycnocline (Sorokin, 2002). This density boundary effectively limits the vertical exchange between the oxygenated upper layer-influenced by the atmospheric and fluvial processes – and the almost completely isolated anoxic deep water. Despite its >2000 m depth, most of the biological activity (apart from bacteria) is hosted within the upper 100–150 m.

The Black Sea is characterized by a positive water balance that results in a net outflow into the Mediterranean. With a drainage basin five times more extensive than the sea area (Ludwig *et al.*, 2009) it works as a virtually isolated ecosystem, and is sensitive to distant anthropogenic activities. This terrestrial influence, together with a contrasting bathymetry and a cyclonic Rim Current (Stanev, 1990), contributes to the Black Sea horizontal zonation (Ragueneau *et al.*, 2002). Two distinct regions can be recognized: the wide and shallow Northwest Shelf (<200 m) and the deep central sea (>1000 m). The latter is mostly isolated from the riverine inflow, which is known to be a key driver on the shelf. Although hydrographic processes, such as mesoscale eddies, filaments, and jets, effectively link these two subsystems together (Zatsepin *et al.*, 2003), they have been seen to show biological differences (McQuatters-Gollop *et al.*, 2008). Productivity of the shelf system appears to be primarily phosphorus limited whereas the open sea system would appear to be nitrogen limited and much more dependent on mixing processes for nutrient supply (Garnier *et al.*, 2002).

Climate affects the Black Sea via atmospheric transfer and riverine inflow. The latter has been demonstrated as a significant factor for the overall water balance and basin-scale circulation (Oguz *et al.*, 1995), as well as nutrient loading from human activities in surrounding land. The Danube River provides about 70% of the freshwater inflow. Thirty-three and 56% of the phosphorus emissions are estimated to be derived from agriculture and urban settlements, respectively; only 8% is considered to be of natural origin (Kroiss *et al.*, 2005). During the 1980s, the Black Sea underwent severe eutrophication caused by economical and lifestyle changes in the surrounding countries, including intensive animal farming and increasing use of agrochemicals and phosphate detergents.

The physical environment of the Black Sea has a major influence across the food web at different time scales (Daskalov, 2003) and has been shown to be influenced by the Atlantic climate through cross-Europe atmospheric teleconnections (Polonsky *et al.*, 1997; Oguz *et al.*, 2006;).

The food web in the Black Sea is relatively simple and effects of both resource (bottom-up) and predation (top-down) have been identified. Major effects of predators at top and middle trophic levels have been found to drive system-wide trophic cascades (Daskalov *et al.*, 2007). The overfishing of pelagic top predators in the 1970s, of planktivorous fish in the 1990s, and the unintentional introduction with ships' ballast water of the ctenophore *Mnemiopsis leidyi* (Konsulov & Kamburska, 1998) resulted in alternating changes in the abundance of the phytoplankton and zooplankton populations, which disturbed the structure and functioning of the entire pelagic food web (Kideys, 2002; Murray, 2005;).

The Black Sea have been populated, exploited, and explored by humans since the antiquity, but major anthropogenic changes such as fish stock collapses, cultural eutrophication, and invasions by alien species have occurred since the 1980s. Initially most changes were attributed solely to cultural eutrophication (Zaitsev, 1993; Bologa *et al.*, 1995;). More recently other factors, such as hydroclimate (Daskalov, 2003; Oguz *et al.*, 2006;), predation effects, and fishing (Bilio & Niermann, 2004; Daskalov *et al.*, 2007;) have been recognized as contributing to the changes.

As put forward above, the recent history of the Black Sea is a combination of abrupt ecological events of great interest to the scientific community. Therefore, this system is an excellent location to study how the marine food web responds to various perturbations that, to varying degrees, occur in the world's oceans. Human activities affect ecological processes in a variety of ways. Harvesting and climate change (Stenseth *et al.*, 2002), for instance, are known to have broad ecological consequences. It is less appreciated that activities in one ecological biome might affect the ecology of another biome. The sensitivity of the Black Sea to human-induced changes in the Danube watershed makes this system an ideal test basin to investigate the effect of socio-economical transformations on the marine biome.

In this study we first address the dynamics of the Black Sea food ladder by estimating an individual model for each of the trophic levels: phytoplankton, zooplankton, gelatinous plankton, and fish. This set of models allows us to empirically study how the terrestrial, climatic, and marine (environmental and trophic regulation) effects influence the food web. The model formulation is tailored to detect and quantify the ecological thresholds at which a given covariate changes its effect on the response variable.

In the second part we combine the previous empirically deduced relationships in one single statistical model. On this basis, the new model reproduces the observed biomasses based only on external drivers and the estimated relationships amongst trophic levels. With the focus on the trophic architecture of the food web, this global food-web model is run under hypothetical scenarios.

Making use of a novel methodology, the present study aims to provide insight on how the marine food web restructures to accommodate changes in the intensity of different pressures (e.g., fishing or eutrophication) and by doing so assess the resilience of the Black Sea ecosystem as its capacity to buffer and withstand disturbance (Holling, 1973; Folke, 2006;).

## Material and methods

### Trophic levels

Previous work has established that cascading trophic interactions can explain the main patterns in the Black Sea time series (Daskalov, 2002, 2003; Daskalov *et al.*, 2007). These interactions are detected across trophic levels and characterize the dominant flows of biomass in the food web.

In the present study the system's food web complexity is compressed into five components, corresponding to four trophic levels: primary producers (phytoplankton), primary consumers (zooplankton), secondary consumers (planktivorous fish, jellyfish), and top predators (piscivorous fish).

Although both planktivorous fish and gelatinous plankton feed on zooplankton, they are considered separately due to their different ecosystem functioning and management implications. Gelatinous plankton comprises *Aurelia aurita* and *M. leidyi* while planktivorous fish includes anchovy (*Engraulis encrasicolus*), sprat (*Sprattus sprattus*), and horse mackerel (*Trachurus mediterraneus ponticus*). Diet spectrum and trophic flow arguments (Shlyakhov & Daskalov, 2008) are at the base of such aggregation, see also Ecopath model in Daskalov (2002).

### Data series

We used annual time series accounting for the various trophic levels (Fig. 1b–e) and environmental variables. The total database consisted of the biomass of phytoplankton (PHY), the biomass of zooplankton (ZOO), the gelatinous plankton biomass (GEL), the planktivorous fish biomass (FIS), fishing mortality (F), the predatory fish biomass (PRE), the sea surface temperature (SST), the North Atlantic Oscillation (NAO) index, and the total inorganic phosphorus loading in the Danube delta (P).

The biological time series were compiled based on data from long-term monitoring collected by the Institute of Fisheries and Aquaculture, Varna (Bulgaria), and the Southern Scientific Research Institute of Marine Fisheries and Oceanography (YugNIRO), Kerch (Ukraine). Data were standardized to zero mean and unit variance, see details in supporting information material in Daskalov *et al.* (2007). This dataset is intended to be representative of the whole Black Sea. This is particularly valid for fish stocks which are estimated using population models applied to data from all Black Sea fisheries (Prodanov *et al.*, 1997; Daskalov *et al.*, 2008;). All plankton components however, might be biased, giving the Northwest Shelf dynamics a proportionally larger weight than the open sea because of the higher productivity and intensity of processes as well as more accurate and frequent sampling along the shelf areas.

Fishing mortality (F) was estimated as the ratio of total catch to biomass of the three dominant species of planktivorous fish in terms of biomass and catches (Prodanov *et al.*, 1997). This index is meant to account for the cumulative ‘trophic’ effect of the fisheries on FIS and through them on other groups such as jellyfish and plankton. Predatory fish biomass (PRE) includes bonito, bluefish and mackerel, all pelagic fish predators mainly feeding on FIS.

The SST time series consists of annual mean values over the whole Black Sea area extracted from the ICOADS dataset published in http://ingrid.ldeo.columbia.edu/SOURCES/.NOAA/.NCDC/.ERSST/.version2/.SST/

The NAO index corresponds to the difference in normalized sea level pressures between Lisbon (Portugal) and Reykjavik (Iceland) over the winter season and was extracted from http://www.cgd.ucar.edu/cas/jhurrell/indices.html

Total inorganic phosphorus loading (P, tonnes) were measured at the Vilkovo station of Kilya branch of the Danube River. Data were compiled and analysed by Daskalov (2003) based on Juravleva & Grubrina (1993) and Weber (1993). Variations in phosphorus loading reflect well human activities in the catchment area (Kroiss *et al.*, 2005) and as such can be considered a proxy for the anthropogenic forcing in the Black Sea system.

### Statistical analysis

The annual averages of the trophic levels' biomasses were used as the response variable and regressed against the various biotic (i.e., the other trophic levels) and environmental variables in the year before. The regression analysis was performed using generalized additive models (GAM) (Hastie & Tibshirani, 1999).

#### Model estimation

To avoid model over-fitting, the number of knots used in each of the GAM splines were kept to a maximum of four. As we were interested in characterizing nonadditive responses in relation only to the relative abundance of the various trophic levels and the environmental conditions, time (‘years’) was not used as a predictor. These precautionary measures and the common model selection procedures (see below) ensure the parsimony of the models and that the simulations are only based on the dynamic structure of the system.

#### Model selection

Model selection was based on a step-wise approach, aimed at removing covariates with a *P*-value >0.05 and minimizing the generalized cross validation (GCV) criterion of the model (Wood, 2000). The GCV is a proxy for the model's out-of-sample predictive performance and it is analogous to Akaike's Information Criterion (Akaike, 1974).

The residuals of the models appeared to be uncorrelated over time and followed a normal and homoscedastic distribution in all cases (Fig. S1), except for the gelatinous plankton model (Fig. S1c). To test the effect of these two outliers on the fitted model, we refitted the model by including two dummy variables accounting for the outliers (see text and Fig. S3 in SI).

#### Threshold GAM

Several regime shifts have been reported in the system (Daskalov *et al.*, 2007) indicating that the food-web interactions and their relationship with the environment might be nonadditive (i.e., different across regimes). To account for regime-dependent relationships, we used a modified GAM formulation, the threshold generalized additive model (TGAM).

This GAM formulation allows for nonadditive effects of the explanatory variables below and above a certain value of a threshold variable (or a combination of variables), i.e., the regression structure is allowed to switch between two GAMs. The threshold is estimated from the data.

#### Detection of regime-dependent dynamics

To compare threshold models (TGAM) with the fully additive model (GAM) formulations (i.e., without threshold) it is necessary to account for the additional parameter used for the threshold search (Ciannelli *et al.*, 2004). The above-mentioned GCV is only a (good) approximation of the real CV and it does not take into account the fact that a grid search has been put in place to find the value of the threshold. Thus, we used the genuine CV to compare models (Table S1), which equals the average squared leave-one-out prediction errors; the leave-one-out prediction is obtained by removing one data case at a time from the model fitting and predicting its value from the resulting model.

#### Sensitivity analysis

CV was also used to assess the predictive performance of the final set of models (see details in supporting information, Figs S4–S8).

#### Simulations

The fitted models were used to simulate the observed dynamics after linking the trophic levels together. Specifically, we used the observations at time *t* to predict the various trophic levels at time *t*+1. Once we got the first prediction for the different trophic levels (at *t*=2), the latter were input as biological variables in the various models to predict the subsequent values at *t*+2, *t*+3, …, *t*+*n*. The covariates were fixed at their observed values. By doing this, we let the food web interact according to the estimated models. Also, as we did not used ‘time’ as a predictor (see above), the simulations are only based on the dynamic structure of the system.

Noise was added to the biotic variables by sampling (with replacement) the model residuals. To preserve the contemporaneous correlation of errors, a whole vector of errors for the four trophic levels corresponding to a randomly sampled year was used at a time. One thousand Monte Carlo simulations were run for each trophic level from which the mean and the 95% prediction bands were calculated.

#### Scenario construction

This skeletal food-web model was afterwards used to investigate the evolution of the system under different conditions (i.e., scenarios). The procedure consisted of three steps: (a) we defined scenarios where some variables were either increased or decreased by a percentage of the mean (e.g., −25%, −15%, +15%, +25%), (b) these modified variables (or scenarios) were input to the various models and, (c) the ‘simulated system’ (i.e., the biomass for the different trophic levels under a given scenario) was investigated with reference to the prevailing food-web control (bottom-up vs. top-down) using phase space plots.

All the models were coded in r (v 2.5.1) (R Development Core Team, 2007) using the TGAM library (created by K.-S. Chan) that relies on the mgcv library (Wood, 2006). All the plots (except Figs 1a and 3) were made with r.

## Results

### Black Sea ecological dynamic structure

**Fig. 2:**
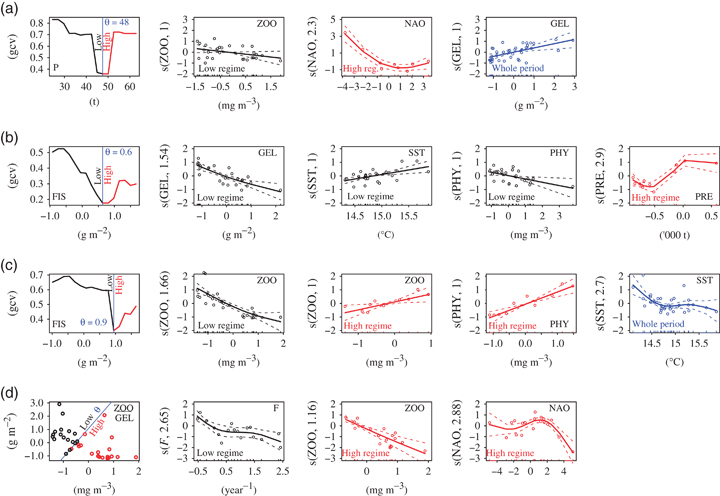
Statistical models. Threshold estimation (first column) and partial plots showing the main biotic and abiotic effects for each of the trophic levels: phytoplankton (a), zooplankton (b), gelatinous plankton (c), and planktivorous fish (d). For the univariate thresholds, phosphorus (a) and fish (b–c), the threshold estimation (generalized cross validation minimization) and threshold value (θ) defining the low (black) and high (red) regime are indicated. For the fish model (d) the blue line (θ) corresponds to the bivariate threshold that assigns the space made by the two variables (zooplankton and jellyfish) to the low (black dots) and high (red dots) regimes. The individual effects are referred either to the low (black) or the high (red) regime of the threshold variables. Those effects acting throughout the whole range of the threshold variable are shown in blue. The *y*-axis indicates the partial additive effect that the term on the *x*-axis has on the response variable. The numbers in parentheses on the *y*-axis indicate the estimated degrees of freedom, which also appear in Table 1. Residuals check (independence, normality, and homoscedasticity) and regime assignation of the actual levels of the threshold variable are shown in Fig. S1.

The most appropriate model structure found for each trophic level is shown below [Eqns (1)–(4)], where 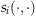 denote nonparametric smooth functions (natural cubic splines) with the first argument enclosed in the parentheses being the covariate and the second argument the estimated degrees of freedom of the splines. The threshold variables and the threshold values delineating the regimes are also given. In the case of bivariate threshold, the regimes are delineated by a line estimated from the data (see Fig. 2d). The residuals showed no serial auto-correlation (Fig. S1) indicating that the following set of models captured most of the system's variability (an average of 70% of explained variance, see details in Table 1 and observations vs. predictions from these models in Fig. 1):

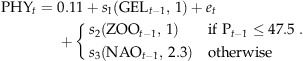
(1)

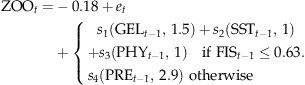
(2)

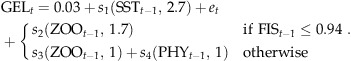
(3)

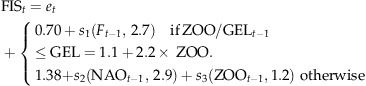
(4)

**Table 1:** Generalized additive models (GAM) models results

	PHY				ZOO		
			
		Estimate	*P*-value			Estimate	*P*-value
			
	Intercept	0.114	0.235		Intercept	−0.178	0.009
	Threshold (θ)	47.52			Threshold (θ)	0.634	
Regime	Covariate	edf	*P*-value	Regime	Covariate	edf	*P*-value
*P* ≤ θ	ZOO	1.00	0.101	FIS ≤ θ	GEL	1.54	< 0.001
*P* > θ	NAO	2.23	< 0.001	FIS ≤ θ	SST	1.00	< 0.001
–	GEL	1.00	0.004	FIS ≤ θ	PHY	1.00	0.002
–				FIS > θ	PRE	2.90	0.017
	*R*^2^ (adj)=0.710				*R*^2^ (adj)=0.816		

	GEL				FIS		
			
		Estimate	*P*-value			Estimate	*P*-value
			
	Intercept	0.033	0.697		Intercept ≤ θ	0.700	< 0.001
	Threshold (θ)	0.944			Intercept > θ	1.378	< 0.001
Regime	Covariate	edf	*P*-value	Regime	Covariate	edf	*P*-value
FIS ≤ θ	ZOO	1.66	< 0.001	Z/G ≤ θ	F	2.65	0.0014
FIS > θ	ZOO	1.00	0.023	Z/G > θ	NAO	2.88	< 0.001
FIS > θ	PHY	1.00	< 0.001	Z/G > θ	ZOO	1.16	< 0.001
–	SST	2.70	0.003				
	*R*^2^ (adj)=0.742				*R*^2^ (adj)=0.628		

Intercept, estimated degrees of freedom (edf) and significance (*P*-value) of the various effects, and *R*^2^ for the four trophic-level models. The threshold values are also reported and whether the effects of the covariates apply to its lower or higher regime is indicated by the notations ≤ θ and > θ, respectively. Note that for the fish model the threshold is defined by a line (intercept: 1.07, slope: 2.18) and not a single value (Fig. 2d). All regimes are defined in terms of the lag 1 of the threshold variables.

These results support that the various trophic levels relate nonadditively to the environment and other trophic levels because the models including thresholds are preferred to their fully additive equivalents, based on CV (Table S1). The nonadditivity consists of the responses switching between two distinct regression functions upon crossing a level (threshold) given by a threshold variable(s) that could be either environmental [Eqn. (1)], biological [Eqns (2) and (3)], or a combination of two biological variables [Eqn. (4)].

GAMs are relatively complex regression techniques in terms of the mathematical formulas behind the smoothers, but are very intuitive when presented pictorially by plotting the graphs of its component functions (Fig. 2). GAMs also enjoy the advantage of being nonparametric (i.e., there is no need to *a priori* specify the functional forms between the response and the explanatory variables). This characteristic gives great flexibility as we let the data tell us what these functional forms look like.

#### Phytoplankton

Phytoplankton showed a nonadditive response corresponding to different levels of the first lag of phosphorus load (Fig. 2a and Table 1). When this was low, the biomass of zooplankton had a slightly negative effect, suggesting that the latter were able to efficiently graze on phytoplankton.

When the levels of phosphorus were high, negative NAO values had a strong effect indicating enhanced climate-driven primary productivity. A positive winter NAO index is associated with cold and dry air masses in southern Europe and the Black Sea region because the westerly winds take a more northwards direction. Conversely, a negative NAO index implies milder winters, with warmer air temperatures and less dry/more wet atmospheric conditions over the Black Sea due to the more direct effect of the Westerlies over the region (Oguz, 2005). Negative NAO years are therefore associated with greater run-off and higher temperatures (Polonsky *et al.*, 1997; Konsulov & Kamburska, 1998; Oguz *et al.*, 2006;). The combination of favourable atmospheric conditions (i.e., negative NAO) and high phosphorus emissions results in increased phytoplankton biomass.

For the whole range of phosphorus emissions, we found a positive effect of gelatinous plankton on phytoplankton suggesting a cascading effect through predation on zooplankton.

Overall the model explained 71% of the variance (see *R*^2^ in Table 1) and the predictions matched very well the observations, not only for the low frequency oscillations but also for the high frequencies. See how the predictions are able to capture most of the observed peaks in Fig. 1b (see also the out-of-sample prediction performance in Fig. S4a). The emissions of phosphorus over the years and residuals check are shown in Fig. S1a.

#### Zooplankton

The dynamics of zooplankton were found to shift between two regimes delineated by the level of the lag 1 of planktivorous fish abundance (Fig. 2b and Table 1). At low fish pressure, increasing levels of gelatinous plankton led to decreasing levels of zooplankton, suggesting a predatory effect. Also, under low planktivorous fish conditions, there was a positive effect of temperature witnessing the existence of bottom-up effects (temperature-related growth) while phytoplankton biomass showed a negative effect reflecting the top-down control of zooplankton on phytoplankton.

For the alternative regime (i.e., with high planktivores) there was a nonlinear but generally positive effect of the predatory fish, indicating an indirect (cascading) top-down effect of the highest trophic level. That the planktivorous fish is found to be the threshold variable controlling the switching of the zooplankton dynamics (Fig. 2b and Fig. S1b) confirms the previous hypothesis that planktivory by fish is a structuring factor in the food web (Daskalov, 2002).

#### Gelatinous plankton

The gelatinous plankton dynamics also alternated depending on the lag 1 of the abundance of planktivorous fish (Fig. 2c and Table 1). If this was low, there was a negative effect of zooplankton, while this effect shifted to be positive when vertebrate planktivores were high.

At high fish abundance, there was also a positive effect of phytoplankton. Phytoplankton cells have been reported to be inside the stomach of *Mnemiopsis* (Tzikhon-Lukanina & Reznichenko, 1991). Whether this is just a consequence of water filtering or does indeed indicate active feeding on phytoplankton is still under discussion. The most likely explanation is that phytoplankton comes in this model as a proxy for climate conditions or eutrophication intensity and does not represent a true tropic interaction (Richardson *et al.*, 2009).

A weak effect of temperature, characterized by low temperatures positively affecting jellyfish, was detected independent of the level of fish. The gelatinous carnivores time series used in this study accounted for both *A. aurita* and *Mnemiopsis leidyi*. The autochthonous *Aurelia* is known to have a competitive advantage over its invasive counterpart *Mnemiopsis* during colder conditions. The partial effect of temperature may have captured the increased contribution of *Aurelia* at the expense of *Mnemiopsis*, for example, during in the colder 1980s (Oguz, 2005).

#### Planktivorous fish

The planktivorous fish dynamics were found to be nonadditive, depending on a bivariate threshold defined by the combined level of zooplankton and gelatinous plankton (Fig. 2d and Table 1). Instead of a single value, the threshold is now defined by a line that divides the space, made up by the zooplankton/gelatinous plankton values, into two regions. These regions correspond broadly to (a) high zooplankton and low jellyfish (favourable conditions, red dots) and (b) low zooplankton and high jellyfish (less favourable conditions for fish, black dots). The region to the right of the threshold line (good conditions) was termed the ‘high regime’ and the alternative ‘low regime’.

In the presence of abundant cnidarians (unfavourable conditions for fish) the fishing mortality was found to be the only explanatory variable.

For the high regime, there was a trophic effect reflecting strong predation on zooplankton (top-down) and a climate effect as positive NAO was associated with low fish abundance.

Positive NAO years correspond to low temperatures over the region and low run-off. Our results show that these conditions have a negative impact on fish, most likely through physiological and life history traits because the trophic effects would be already captured by the additive effect of zooplankton. These two effects were found for the same regime (high zooplankton/low jellyfish) indicating that fish are sensitive to climate only when their food conditions are good (similarly to what was found for phytoplankton).

### Black Sea food-web trophic interactions

**Fig. 3:**
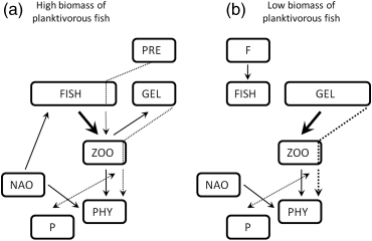
Food-web regulations. Schematic representation of the main trophic interactions under high (a) and low (b) biomass of planktivorous fish, which roughly coincides with the opposite state for gelatinous plankton. Arrows pointing upwards represent resource control (positive effect between consecutive trophic levels). Arrows pointing downwards represent predator control (negative effect). Cascading effects are represented by dashed lines crossing through a trophic compartment. The threshold effect of phosphorus on the phytoplankton dynamics is represented by an oblique dashed line.

While recognizing that statistical relationships do not necessarily imply causality, consistently positive or negative associations between consecutive (or not) trophic levels provided us with useful information about the trophic regulation. The conceptual model presented in Fig. 3 is based on the previous results (Fig. 2), which are interpreted in the following fashion. Given a specific trophic level as response variable, a positive effect of the next lower trophic level is interpreted as bottom-up (resource effect). A negative effect of the next upper trophic level indicates predation. If the predator consumption implies top-down control on the prey this is usually reflected by a negative effect of the prey in the predator model (e.g., phytoplankton and zooplankton, and zooplankton and jellyfish). The fish model was the only one where the top-down effect on zooplankton (negative effect of zooplankton on fish) was not backed by a negative effect of fish on zooplankton. A positive effect of the second upper trophic level is interpreted as a cascade effect (e.g., jellyfish on phytoplankton). Also, a cascade effect of piscivorous predators was reported for zooplankton, indirectly supporting zooplankton consumption by fish.

The schematic representation of Fig. 3 shows the regulatory dynamics under dominance of fish/jellyfish can be assumed to alternate over time. Jellyfish affected negatively the zooplankton when fish biomass was low (Fig. 2b). Zooplankton had opposite effects in the jellyfish model (Fig. 2c): it had a negative effect on jellyfish abundance when fish biomass was low (top-down, downwards thick arrow pointing downwards in Fig. 3b) and a positive effect in the high fish regime (bottom-up, upwards thin arrow in Fig. 3a). Top-down control of zooplankton by fish occurs when the jellyfish biomass is low (Fig. 2d, represented by a thick arrow pointing to zooplankton in Fig. 3a). In contrast to jellyfish, we found no evidence of bottom-up effects of zooplankton on fish. This missing effect can be, however, inferred from the positive effect of zooplankton on predatory fish (Fig. S2).

### Simulation of the Black Sea food web

**Fig. 4:**
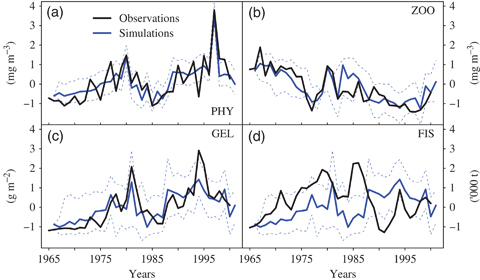
Model simulations. Observations (black) and simulations (blue) of the biomasses of phytoplankton (a), zooplankton (b), gelatinous plankton (c), and planktivorous fish (d). The solid blue line represents the mean and dashed lines correspond to the 2.5 and 97.5 percentiles of the 1000 Monte Carlo simulations.

Figure 4 shows the observations vs. the simulations. The ‘simulations’ or ‘joint predictions’ (blue lines) are obtained by linking trophic levels together while the ‘predictions’ (Fig. 1, red lines) are estimated independently of the other models, simply by predicting the response variable on the observed covariates (see ‘Material and methods’).

The relatively low *R*^2^ of the planktivorous fish model (63%) affects the quality of the simulation of the long-term dynamics when coupled to the other trophic levels (also detected in the sensitivity analysis, Fig. S4d). The possible implications of this problem were addressed by double-checking the simulated scenario results (where fish was simulated) with alternative model runs that used fish observations instead (see SI). The relatively low *R*^2^ of this model shows the difficulty of finding good predictors for the fish biomass data. It may also have to do with the fact that small pelagics (e.g., anchovy) migrate seasonally (Chashchin, 1995) and so can experience different conditions than ubiquitous organisms like jellyfish.

### Scenario results

Once the models were estimated and the simulations succeeded to reproduce the observations (except for the planktivorous fish mentioned above), we used them to explore what would have happened if conditions had been different. We focused on key variables that could cascade up and down in the food web in order to track their effects. In particular we chose to modify phosphorus and planktivorous fish because they affect the system from opposite directions.

#### Phase space

**Fig. 5:**
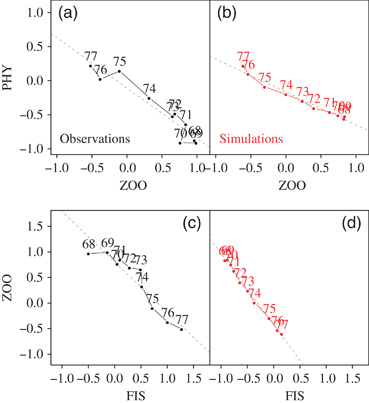
Observations vs. simulations phase space plots. Phase space plots of consumer (driver) against resource (response) during the major ecosystem regime shift of the 1970s. The observations (left column) are shown in black while the simulations (right column) appear in red. Standardized data from Fig. 4 are used. Numbers on the plots are years.

Phase space plots of consumers (as drivers) against resource (response) were used by Daskalov *et al.* (2007) to explore the causality behind the shifts reported in the Black Sea in different periods. Low-resource/high-consumer indicating dominant top-down control and vice versa. Figure 5 shows these trajectories during the major regime shift of the 1970s for both phytoplankton/zooplankton and zooplankton/fish. The simulations mirrored the observed trajectory consisting of a linear increasing trend over the years for the first two trophic levels (Fig. 5a and b) and a linear, but decreasing, trend for the next couple of links (Fig. 5c and d). These two divergent patterns reflect the trophic cascade that the depletion of predatory fish triggered, which caused the restructure of the whole food ladder; increase of planktivorous fish, decrease of zooplankton, and increase of phytoplankton (Daskalov *et al.*, 2007). Here, we used the same approach to investigate changes in the trophic control over time under the various scenarios. These trajectories were simplified to better illustrate trophic shifts. In particular, for this first major regime shift of the 1970s, the trajectories were assimilated to straight lines and the changes in the slopes compared (the approach for other regime shifts and further details are given in SI).

### Understanding the regime shifts

**Fig. 6:**
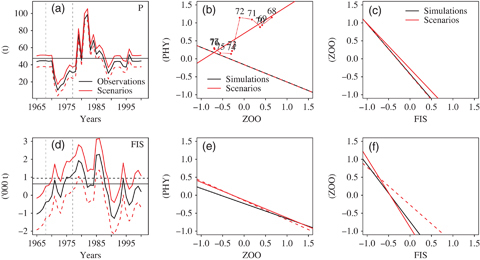
Prevailing food web control under different scenarios. The plots on the left column (a and d) show the phosphorus and planktivorous fish levels (i.e., observations and scenarios). The horizontal lines are the threshold values of the models for phytoplankton (a), and zooplankton and gelatinous plankton (d, continuous and dashed lines respectively). The vertical dashed lines comprises the period of time investigated (1970s' shift). The plots to the right (b and c and e and f) illustrate the phase space trajectories (as line slopes) for zooplankton and phytoplankton (b and e), and planktivorous fish and zooplankton (c and f) corresponding to the following scenarios: a 15% of the mean increase and decrease for phosphorus (continuous and dashed red line respectively) and a 25% of the mean increase and decrease for fish. Black lines are observations. The colour and line styles in the phase space correspond to those for the scenarios. In plot b the actual phase space points (years) for the +15% phosphorus regime (from which the slope is estimated) are shown [for a complete view of all the scenario runs and the 1990s shift see Figs S9–S11 and S13–S14].

As explained above, a sequence of rich and poor phosphorus scenarios was entered as free covariates in the empirically deduced skeletal food-web model (Fig. 6a). No changes were found when decreasing the phosphorus loading whereas a slight increase of just 15% was enough to shift the zoo/phytoplankton trajectory (Fig. 6b, see Fig. S9 for all scenarios). A close look at the phytoplankton model structure results [Eqn. (1)] might give us some clues about the mechanisms behind such a shift.

The dynamics of phytoplankton turned out to be nonadditive depending on the level of phosphorus (Fig. 2). Interestingly, under high P emissions, negative NAO was found to enhance phytoplankton biomass while under low conditions this climate proxy had no significant effect. In the late 1960s/early 1970s there were several negative NAO events that had no effect on phytoplankton because phosphorus was below the threshold at the time. By elevating its level above the threshold (i.e., assuming human activity to have been higher) climate (through NAO) was allowed to positively affect phytoplankton, creating new initial conditions before the outburst of planktivores. In these circumstances, the evolution of the zoo/phytoplankton phase space trajectory over the years flipped sign suggesting that this cascading effect would not appear in a case of a higher nutrient enrichment. No remarkable changes were observed between zooplankton and fish suggesting that this effect would, however, not have been able to propagate upwards in the food web (Fig. 6c). Even though the observed changes affected the first trophic levels and did not involve fish they were further verified with fish observations (Fig. S10).

The same procedure was repeated but now changing the biomass of planktivorous fish (Fig. 6d). In this case, no remarkable changes were detected in the trophic regulation during the 1970s for either trophic level (Fig. 6e and f and Fig. S11). A similar phase space approach was taken to explore the resilience of the trophic regulations in the second major shift in the early 1990s (Fig. S12). No drastic shifts were observed under the various phosphorus or fish scenarios (Figs S13 and S14).

## Discussion

As a ‘natural laboratory’, the Black Sea is a very attractive system for studying ecological concepts, such as regime shifts and cascading effects (Strong, 1992; Scheffer *et al.*, 2001; deYoung *et al.*, 2008;). Previous works have focused on the implementation of dynamic ecosystem models (Daskalov, 2002; Gücü, 2002; Lancelot *et al.*, 2002; Oguz *et al.*, 2008;) that are useful for assessing several interesting hypotheses on the underlying mechanisms. More recent research is taking a multidisciplinary approach to simultaneously integrate the social and ecological sides and so assess the implications of alternative development paths on the Black Sea (Langmead *et al.*, 2009). There is still a need to understand how the trophic levels interact with each other and the capability of these interactions of accommodating external pressures by self-organizing. Here, we present a new approach that investigates the structuring forces within the Black Sea food ladder directly from the data. Based upon the Threshold GAM, we characterized the steady state dynamics and regimes of the system. The resilience of the empirically deduced regulatory forces between trophic levels and its propagation through cascading effects were studied by simulation experiments.

The main advantage of the statistical TGAM modelling developed in this study is the compression of complex ecosystem dynamics into a simple set of equations containing a minimum of four primary variables. Despite its simplicity (both conceptual and computational) the TGAM allows for nonlinear and nonadditive responses. These properties ensure a more flexible approach than parametric or fully additive traditional statistical techniques and makes it particularly suited to investigate systems where alternative regimes have been described, such as the North Sea (Beaugrand, 2004) or the Baltic Sea (Möllmann *et al.*, 2009). All these features allow empirically exploring ‘real’ data and quantitatively resolving multiple time-series for regime-dependent dynamics and ‘tipping’ points.

Since the 1960s, the abundance of planktivorous fish progressively increased following the sharp decline of pelagic predatory fish. As a response to increased planktivory, zooplankton decreased and so did their grazing pressure on phytoplankton. Predator release, along with nonlimiting nutrient conditions, made phytoplankton more sensitive to climate (Daskalov, 2002; Oguz & Gilbert, 2007;). Our results indicate that the combination of favourable climatic influences (indexed by negative NAO), nutrient enrichment from land-based sources, and low grazing pressure on phytoplankton resulted in intense eutrophication (Fig. 2a), including the development of massive algal blooms events (red tides) reported during the 1980s (Zaitsev, 1993; Bologa *et al.*, 1995;). Analyses of anthropogenic influence including nutrients, plankton, benthos, bottom hypoxia and hydrogen sulphide production (Zaitsev, 1993; Bologa *et al.*, 1995; Daskalov, 2002, 2003) has shown that eutrophication took place mainly during the 1980s. Modelling studies also suggest that the increase in primary productivity (driven by eutrophication) cannot produce the observed structural changes (trophic cascades) alone, but reduced predation (due to removal of top-predators by overfishing) is the main driver of such changes (Daskalov, 2002).

Our simulations indicate that the removal of secondary carnivores caused a total loss of resilience which pushed the food chain into an alternative state (Fig. 5). As a result, the whole food web reorganized, from primary carnivores all the way down to autotrophs. The food web truncation shows up as a key element of destablization. No matter the biomass of primary carnivores in the system, it would have inevitably turned to the less desirable state of high phytoplankton. According to our scenarios, no management measure on the ‘upgraded’ trophic level (e.g., increase of fishing effort on small pelagics) would have succeeded in counteracting the top-down force (Fig. 6e).

Only at the base of the food web, the interaction between climate (NAO) and fertilization (P) could have partially offset these effects. Our scenarios suggest that by enhancing the bottom-up forces, the trophic reorganization could have given a partly different result. Favourable climate conditions would have remarkably increased phytoplankton much earlier, at the end of the 1960s, allowing zooplankton to better adapt to its resource at a time when the biomass of planktivores (both fish and jellyfish) was still low. Initial conditions characterized by both high autotrophs and herbivores would have made the evolution of these first trophic levels show a different trajectory, resulting in a simultaneous decrease. Runaway consumption by fish (before jellyfish bloomed) would still have been able to graze down zooplankton over the years (although not so smoothly, see points in Fig. 6b). The end result is practically the same for phytoplankton but predicts slightly lower biomass of zooplankton and higher fish biomass. This suggests that under a scenario of early eutrophication, the subsequent reorganization of the food web– caused by the triggering of the trophic cascade– would have resulted in a more efficient transfer of energy (i.e., increased bottom-up effects). These enhanced bottom-up effects would not be detectable above the zooplankton level.

Zooplankton appears as a key trophic level where the bottom-up (human activity on land and climate) and top-down forces (planktivory and predation) converge. Our scenario results show that this trophic level is able to buffer bottom-up effects by changing its trophic interaction with phytoplankton.

The increased abundance of fish planktivores together with the emergence of gelatinous planktivores in the early 1980s contributed to the establishment of a potent level of primary carnivores, which tightly controlled the abundance of zooplankton since the late 1970s. This consumption force has, however, different implications whether exerted by fish or jellyfish. Gelatinous carnivores are able to benefit from zooplankton even under the dominance of its vertebrate competitors, while fish could not get by in the same way when gelatinous plankton dominates. According to the size selective feeding hypothesis (Daskalov, 2002), intense grazing of planktivorous fish eliminates larger zooplankton allowing for better growth of small zooplankton which favours jellyfish development. Field data also suggest that the impact of *Mnemiopsis* is stronger on small zooplankton (Anninsky *et al.*, 1998). The bottom-up effects (Fig. 2c) could therefore be explained by the increase of small zooplankton. Gelatinous plankton have a tighter control on zooplankton as compared with fish and therefore they have a competitive advantage (Fig. 3). *Aurelia* and *Mnemiopsis* have been reported to consume fish eggs and larvae occasionally in the Black Sea. However, this pathway is not considered as energetically or interactively important by most experts (Daskalov, 2002 and references therein). Our results did not detect any direct interaction between these two groups of planktivores but through competition for zooplankton food.

While cnidarians have comparably fewer predators (dead end), human influence directly affects fish from above, making fishing even more important when jellyfish dominates. In sum, planktivorous fish seems to be more vulnerable to perturbations in the system –either driven by climate or human activities – than gelatinous carnivores, particularly when the latter are numerous.

Regime shifts have been described for several European systems around the late 1980s (Alheit & Bakun, 2010; Conversi *et al.*, 2010;). This synchrony suggests a common external driver and the NAO has been proposed as possible large scale climate link. Our modelling approach avoided intentionally using time (year) as explanatory variable as our goal was to reproduce the observed changes based only on the trophic regulation and environmental effects. From this perspective, aspects such as ‘timing’ and ‘synchronies’ are difficult to address. However, the fact that the NAO was found to affect the phytoplankton and fish dynamics suggests that this could be a link for the adjustment of the Black Sea with the neighbouring North, Baltic, and Mediterranean Seas.

The dataset analysed here consists of annual time series integrating the system's spatial and seasonal variability over the last 36 years (Daskalov *et al.*, 2007). While it has the advantage of capturing the big picture, its coarse spatio-temporal resolution may miss some details. Although for most of the groups the explained variance was large it is possible that the deduced relationships vary regionally (Lancelot *et al.*, 2002). As mentioned earlier, this could be the reason behind the low explanatory power of the fish models. As active swimmers, pelagic fish have a greater ability to choose favourable environmental conditions that need not coincide with those averaged for the whole Black Sea. This could be also explained by the relative bias of plankton series which are more representative of the Northwest Shelf compared with fish data which better reflect the average state of the stock in the whole sea. Future research should consider and explore the existence of differential responses of the system to the same drivers, whether on the shelf or the open sea (Ragueneau *et al.*, 2002; McQuatters-Gollop *et al.*, 2008;).

The concept of ‘ecosystem based fisheries management’ (Cury *et al.*, 2008) encourages the consideration of food-web responses (including regime shifts) to climate variability and human pressure in an integrative management of marine resources. Eutrophication (bottom-up) and trophic cascade (top-down) have distinct disturbing effects, which in combination with climate, can greatly deviate the system from a given stable state. Historically, the fisheries-driven trophic cascade first disturbed the structure of the system from above. An already decapitated food web was further degraded by eutrophication. Our results demonstrated that increased productivity could have been more efficiently handled by a more complex (including viable top-predators) and therefore more resilient system. For that reason, a recovery of the previous four-tiered architecture by rebuilding the top-predators could improve the system's ability to counterbalance fluctuations driven by climate or eutrophication. Although a reversal of an ecosystem to the exact previous state is a highly unlikely event (Oguz & Velikova, 2010), some potential for partial recovery have been observed in the Black Sea (Lancelot *et al.*, 2002). Whether the Black Sea will continue its current way to recovery or will return to its highly eutrophic state will depend, to a large degree, on social-economic choices (Langmead *et al.*, 2009). Even in the most optimistic scenario, the Black Sea will never come back to the pre-1960s state after the introduction of *Mnemiopsis*. The question arises as to whether the reconstruction of the food web will decrease the competitiveness of the small pelagics over the gelatinous newcomers.

## Supporting Information

**Figure S1.** Thresholds and residuals. Regime assignment and residuals for each of the four individual models: phytoplankton (a), zooplankton (b), gelatinous plankton (c), and planktivorous fish (d). The first column (a–c) shows the temporal evolution of the threshold variables (phosphorus, fish, and fish, respectively) and their regime allocation: those points above the threshold (represented by the blue line, θ) are shown in red (high regime) while those below appear in black (low regime). The bivariate threshold of the fish model (d) is explained in Fig. 2 (see legend there for details). The following four columns show the inspection of residuals for the assumptions of independence (autocorrelation function), normality (Q-Q plot), and homoscedasticity (residuals vs. time and residuals vs. fitted values). Apart from visual inspection, Shapiro (normality) and Breusch-Pagan (homoscedasticity) tests were performed (values not shown). As commented earlier (see text), only jellyfish residuals violated the latter assumptions.

**Figure S2.** Predatory fish model. Plots showing the effect of zooplankton on predatory fish (a), observations vs. predictions (b), and residual statistics: autocorrelation (c) and normality (d).

**Figure S3.** Alternative gelatinous model partial effects and residuals. Plots showing the estimation of the threshold value (a), all the partial effects (b–e), regime assignment to threshold variable (f), residual statistics (g–h), and predictive performance (i) for the gelatinous plankton model when accounting for the two outliers detected in the residuals.

**Figure S4.** Observations (black) and out-of-sample predictions (purple) of the biomasses of phytoplankton (a), zooplankton (b), gelatinous plankton (c), and planktivorous fish (d).

**Figure S5.** Threshold estimation (a) and partial plots (b–d) for the phytoplankton cross validation models. Each plot consists of the overlaid results from the 36 subset models. The numbers in parentheses on the y-axis indicate the averaged estimated degrees of freedom. See also Fig. 2 legend for further details.

**Figure S6.** Threshold estimation (a) and partial plots (b–e) for the zooplankton cross validation models calculated as for PHY (see legend in Fig. S5).

**Figure S7.** Threshold estimation (a) and partial plots (b–e) for the jellyfish cross validation models calculated as for PHY (see legend in Fig. S5).

**Figure S8.** Threshold estimation (a) and partial plots (b–d) for the fish cross validation models calculated as for PHY (see legend in Fig. S5).

**Figure S9.** 1970s phase space plots for all the phosphorus scenarios. Illustration of the phase space trajectories for zooplankton and phytoplankton (a–b), and planktivorous fish and zooplankton (c–d) for all the phosphorus regimes during the 1970s major shift. The lower phosphorus regimes are shown to the left (scale of reds) and the higher to the right (greens). The legend shows the value of the slopes to the corresponding percentage of the mean increase or decrease.

**Figure S10.** 1970s phase space plots for all the phosphorus scenarios (fish observations). Same as Fig. S9 but using observations for fish biomass, not simulations.

**Figure S11.** 1970s phase space plots for all the fish scenarios. As Fig. S9 but for fish regimes, lower abundance to the left (reds) and higher to the right (greens).

**Figure S12.** Observations vs. simulations phase space plots. Phase space plots of consumer (driver) against resource (response) for the observations (left column, in black) and simulations (right column, in red) during the 90s regime shift. Standardized data from Fig. 4 are used. Numbers on the plots are years. Dashed lines are the slopes of the linear regression lines joining all the points.

**Figure S13.** 1990s phase space plots for all the phosphorus scenarios. Illustration of the phase space trajectories for gelatinous plankton and zooplankton (a–b), and zooplankton and phytoplankton (c–d) for all the phosphorus regimes during the 1990s second major shift. The lower phosphorus regimes are shown to the left (reds) and the higher to the right (greens). The legend shows the value of the slopes to the corresponding percentage of the mean increase or decrease.

**Figure S14.** 1990s phase space plots for all the fish scenarios. As Fig. S13 but for fish regimes, lower abundance to the left (reds) and higher to the right (greens).

**Table S1.** gCVs. Genuine Cross-validation scores (gCV) for the non-additive GAM model (Threshold GAM) and the fully additive equivalent for all the trophic levels.

**Table S2.** Predatory fish (PRE) models results. Intercept, estimated degrees of freedom (edf) and significance (*P*-value) of the covariate, and *R*-squared (*R*^2^) for the predatory fish model (see Fig. S2). It also includes a dummy variable accounting for the effect of an outlier (o1).

**Table S3.** GEL models results – accounting for outliers. Intercept, estimated degrees of freedom (edf) and significance (*P*-value) of the various effects, and *R*-squared (*R*^2^) for the gelatinous plankton model when including two dummy variables (o1 and o2) targeting the two outliers observed in the residuals (Fig. S1c). This model summary corresponds to Fig. S3.
