# Supplementary figures and images for: Overfishing of top predators eroded the resilience of the Black Sea system regardless of the climate and anthropogenic conditions
Source: Glob Chang Biol. 2011 Mar;17(3):1251–65. doi: 10.1111/j.1365-2486.2010.02331.x (PMC3597262; doi:10.1111/j.1365-2486.2010.02331.x)

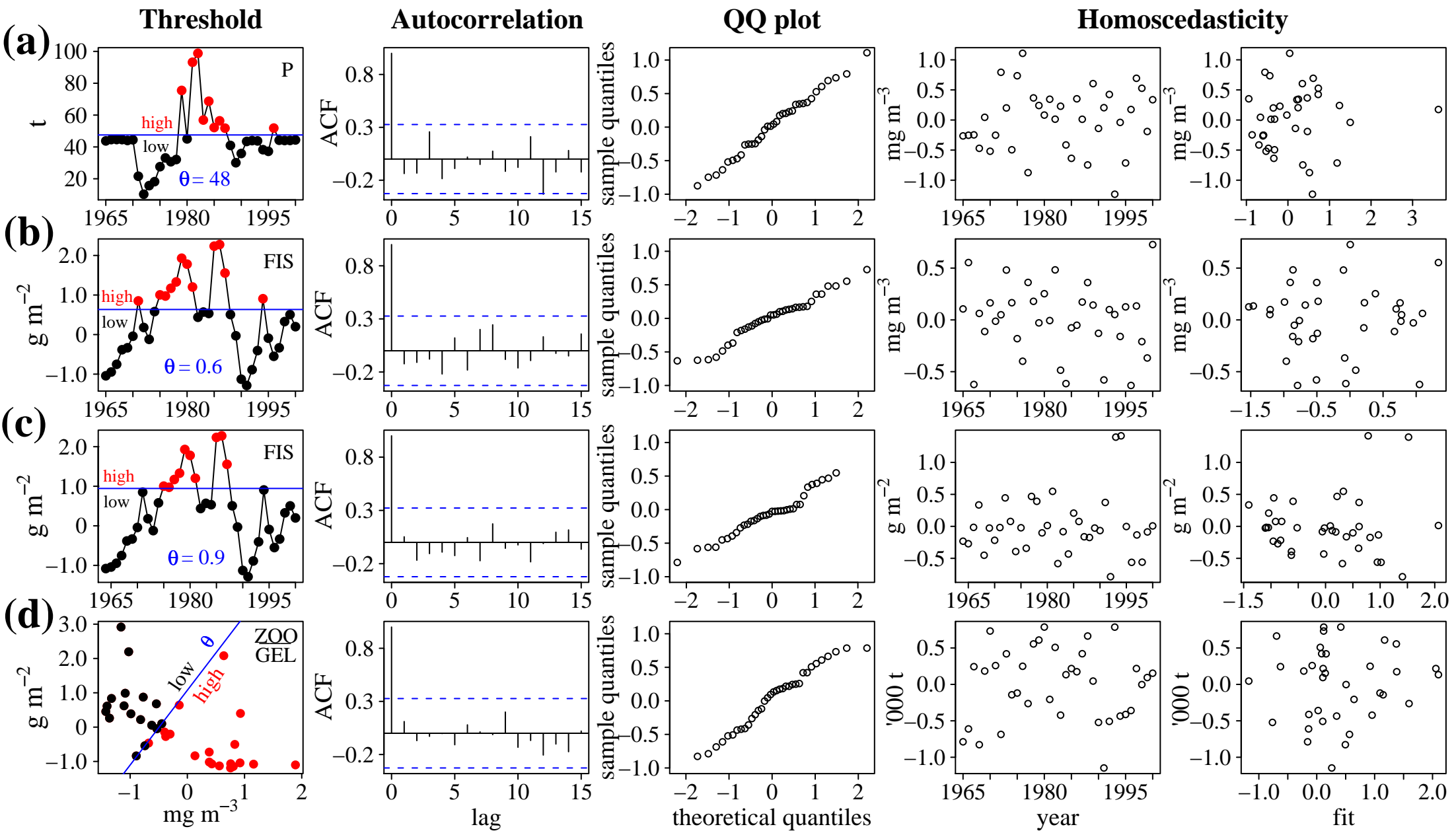

Supplement: Supplementary file 1 [file gcb0017-1251-SD1.pdf]

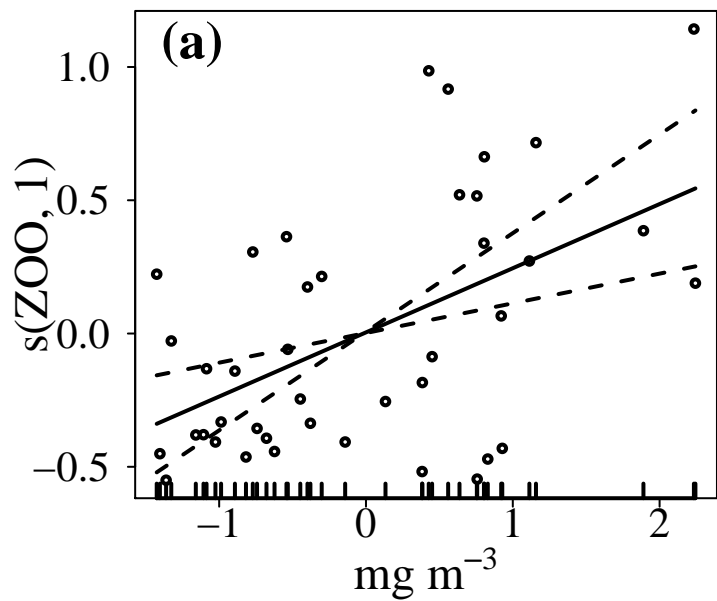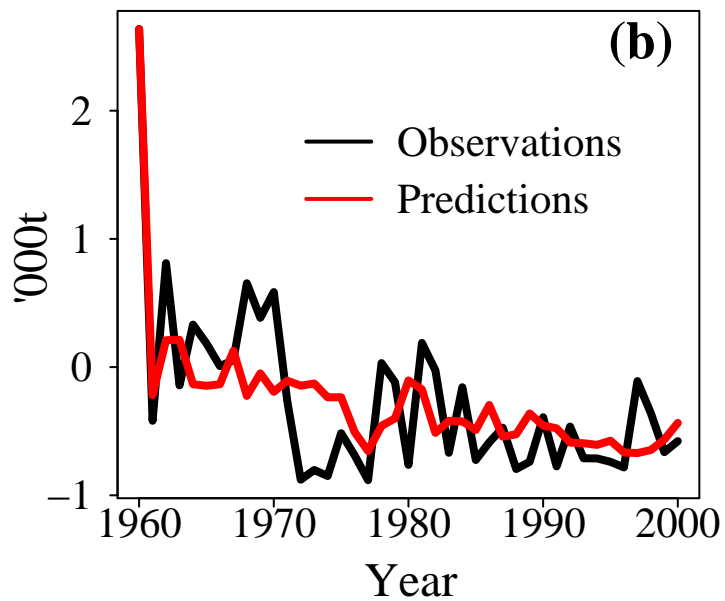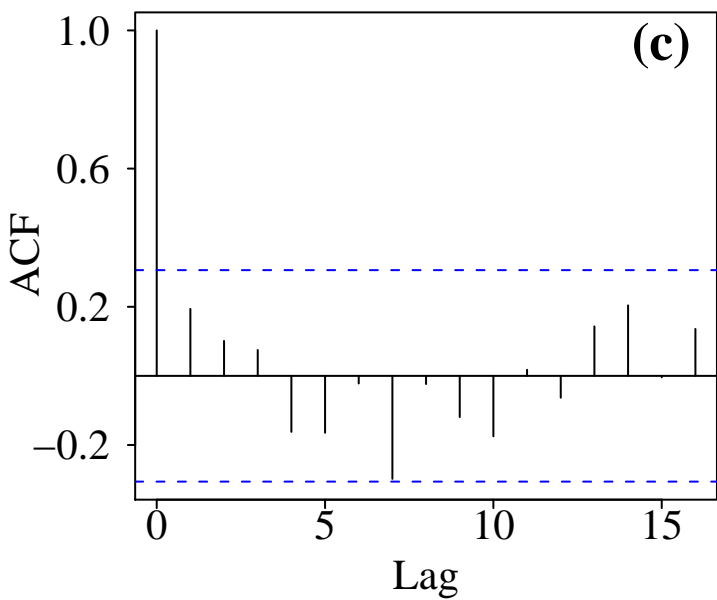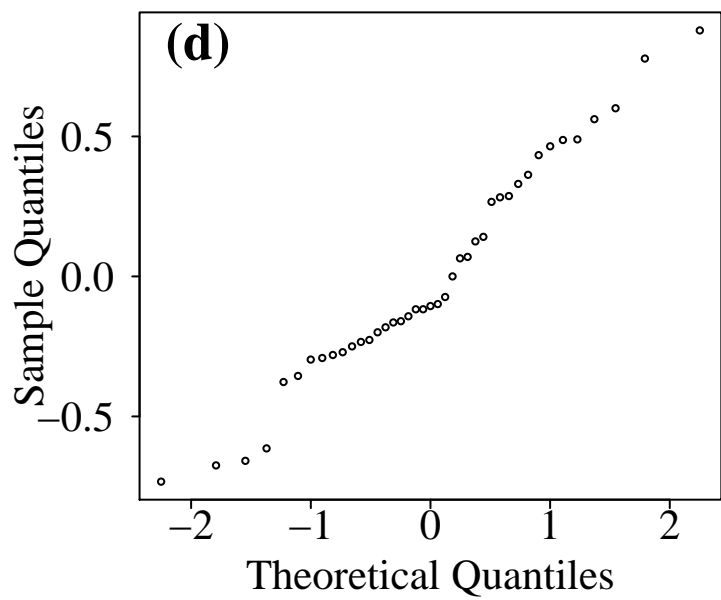

Supplement: Supplementary file 2 [file gcb0017-1251-SD2.pdf]

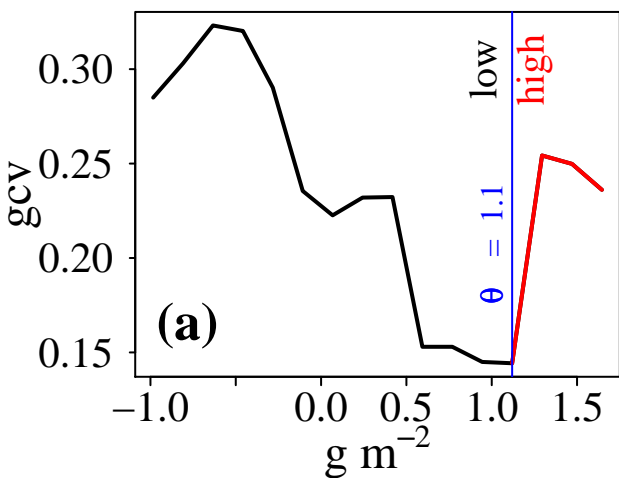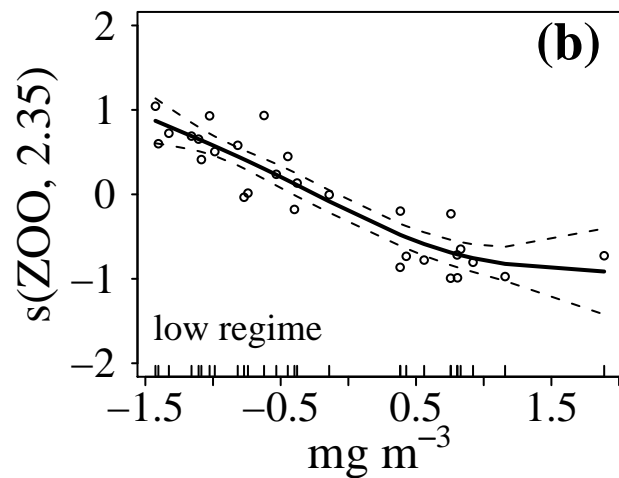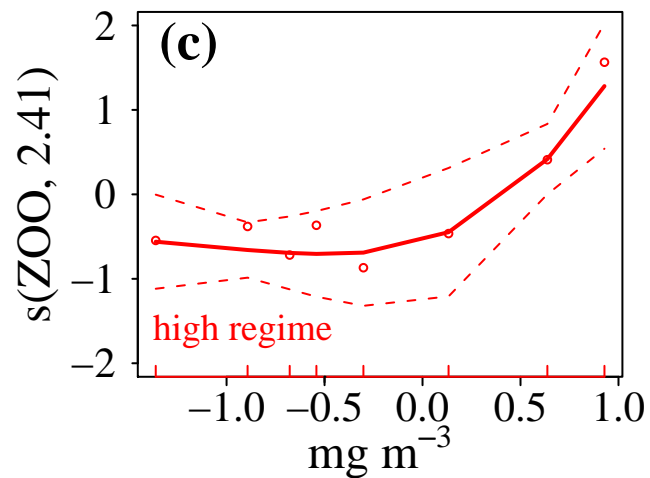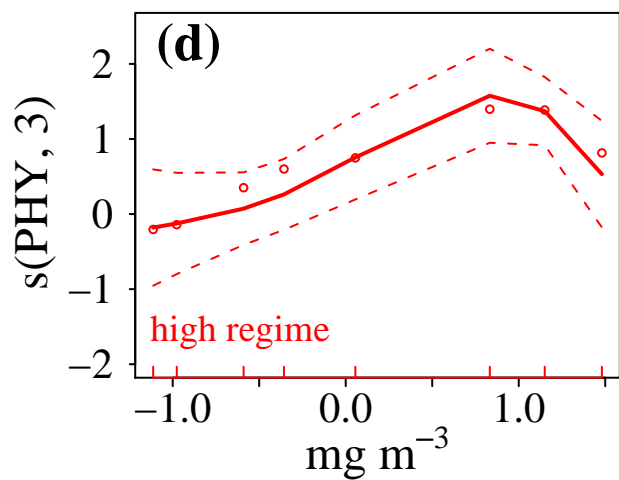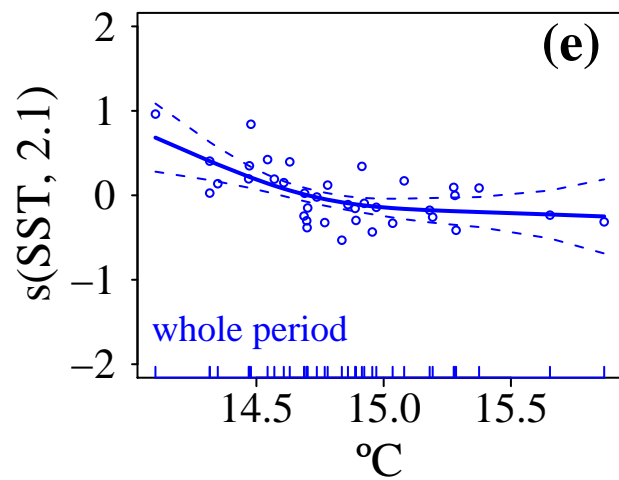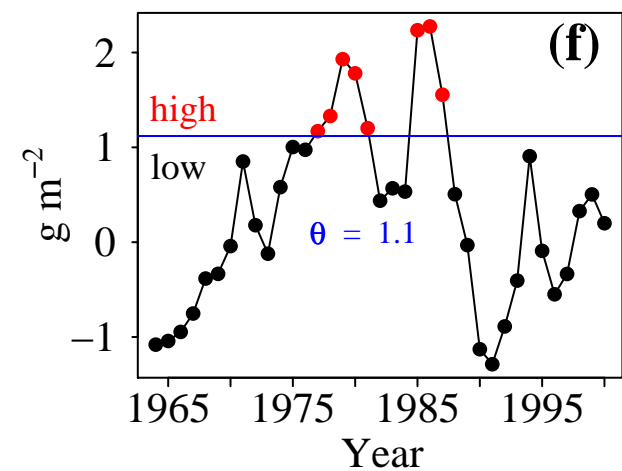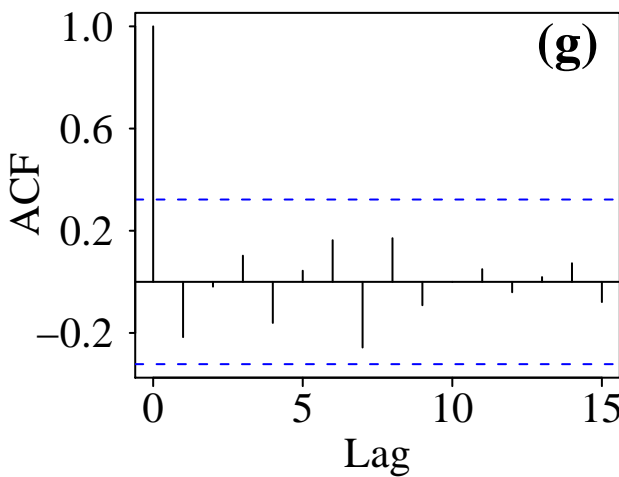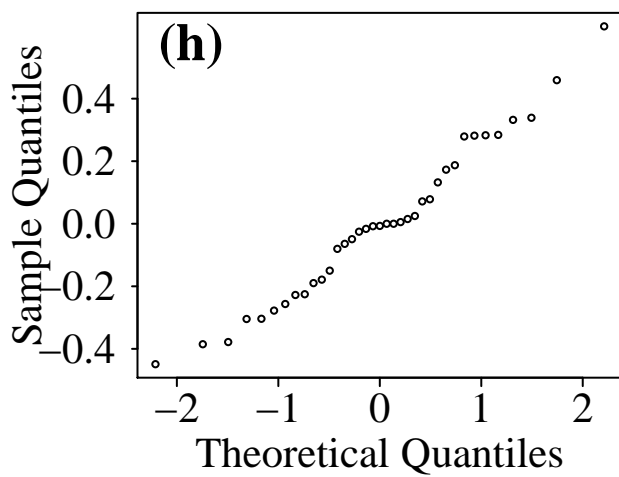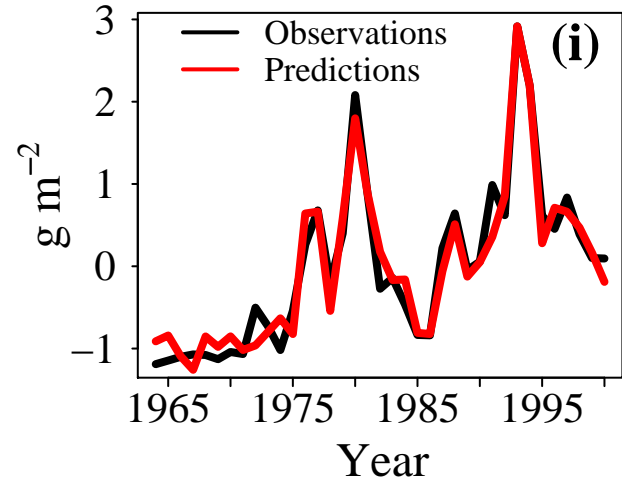

Supplement: Supplementary file 3 [file gcb0017-1251-SD3.pdf]

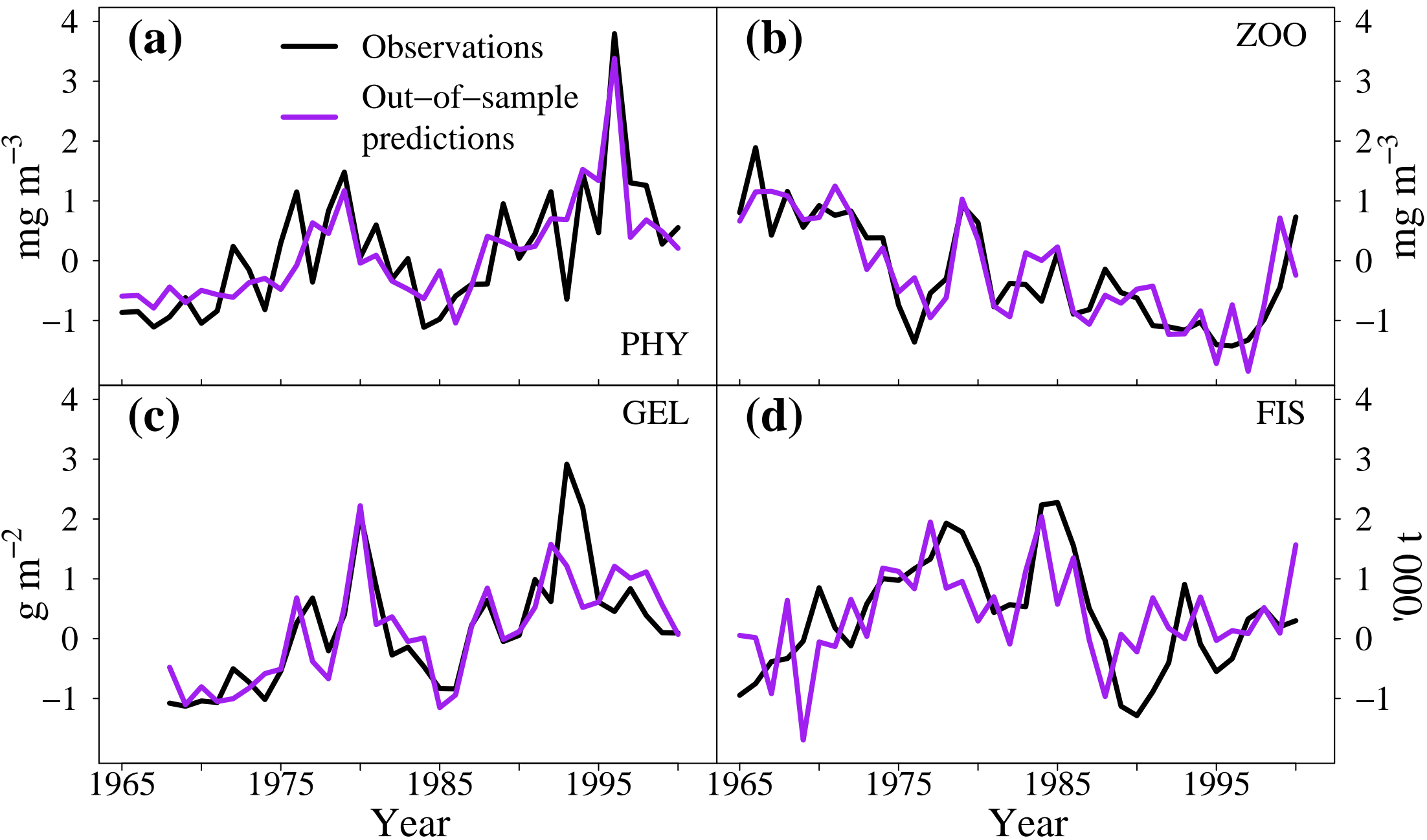

Supplement: Supplementary file 4 [file gcb0017-1251-SD4.pdf]

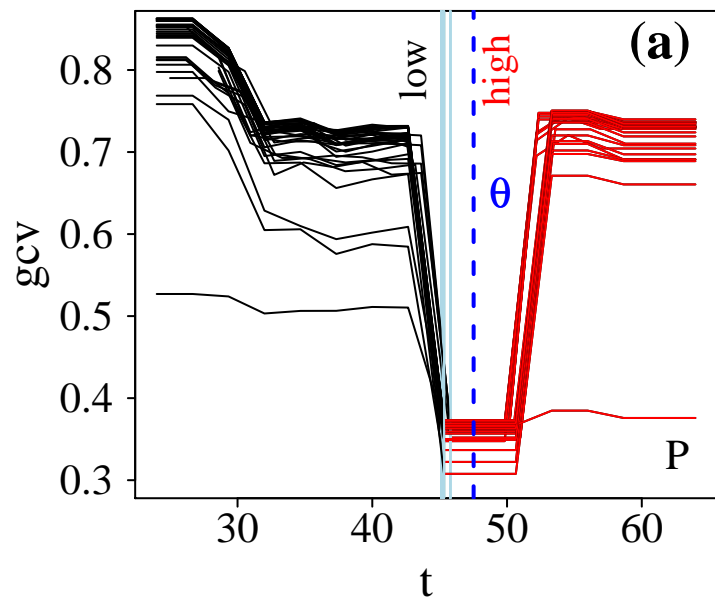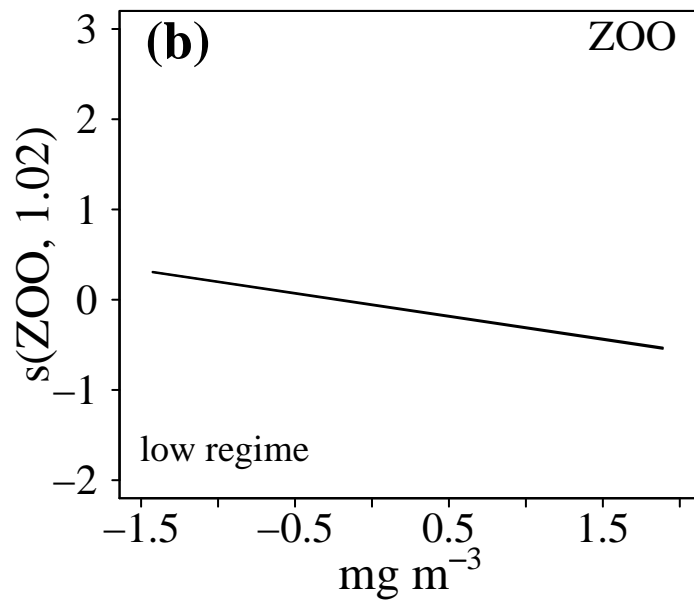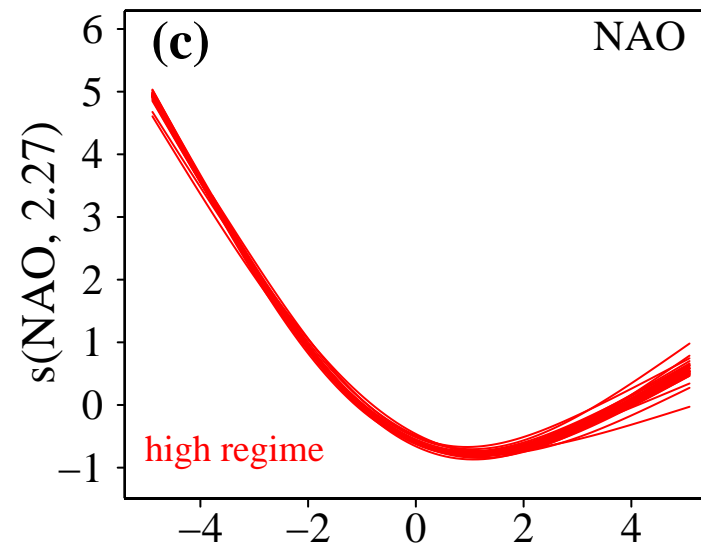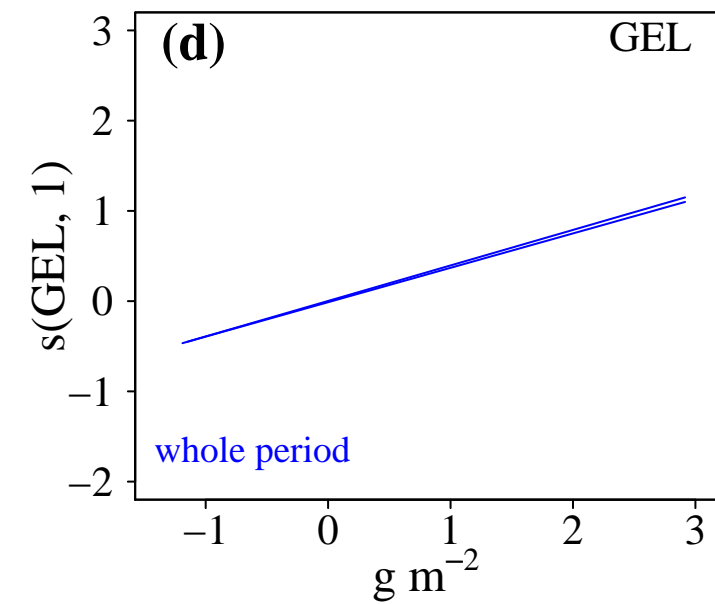

Supplement: Supplementary file 5 [file gcb0017-1251-SD5.pdf]

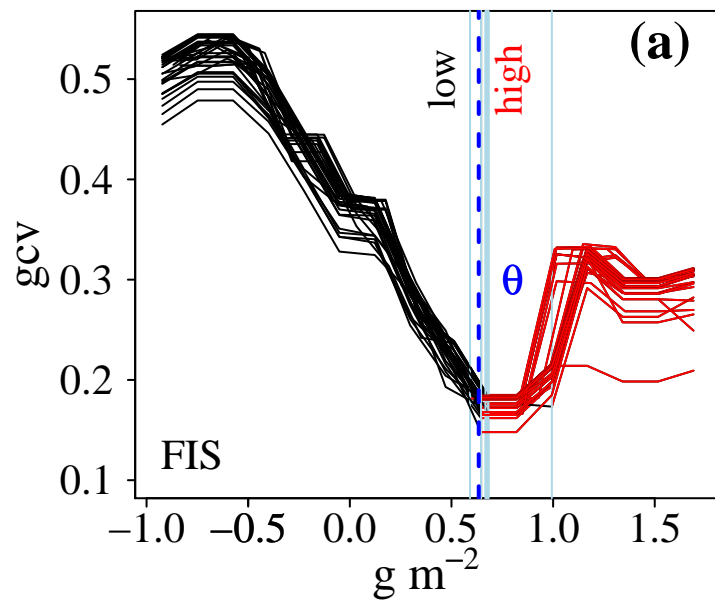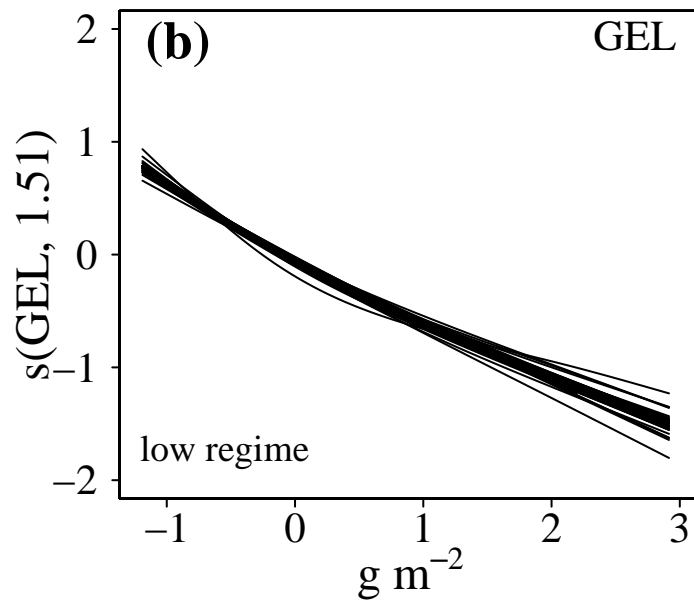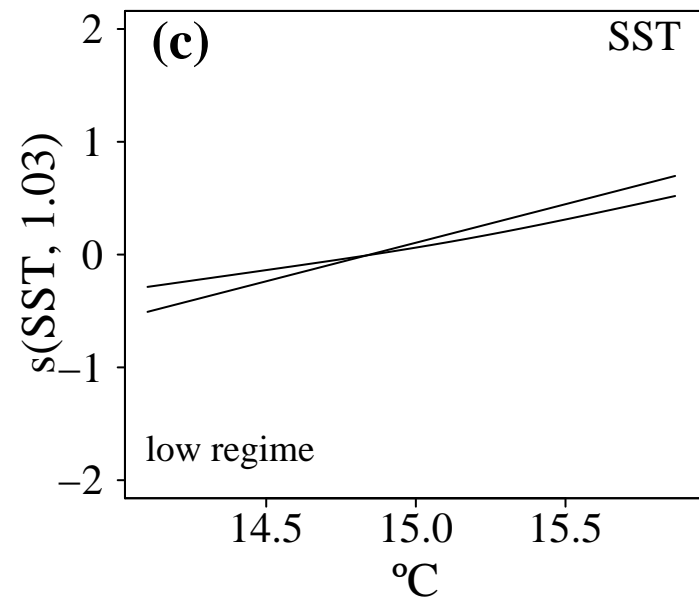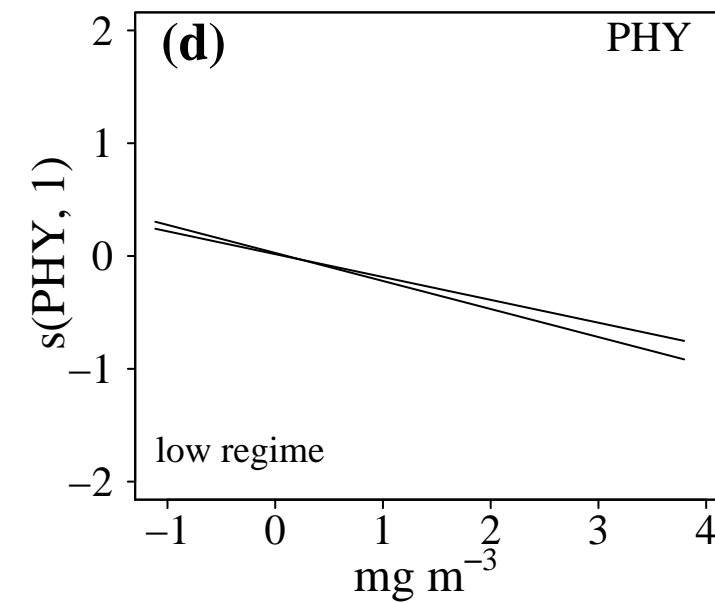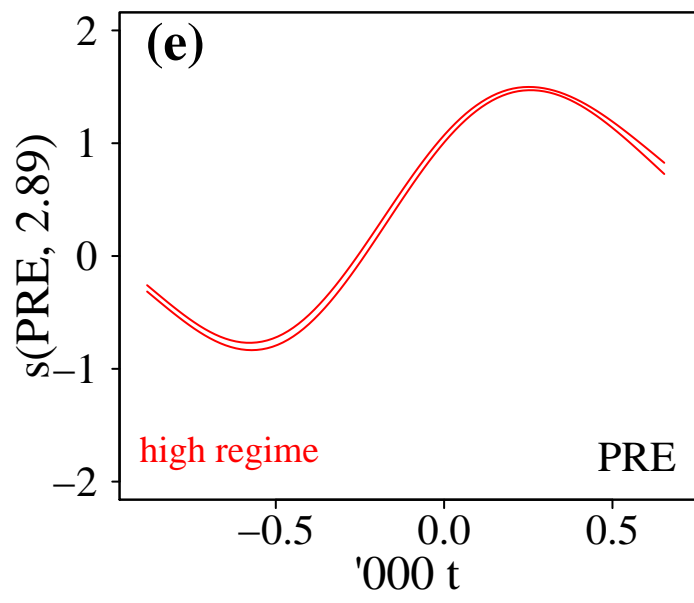

Supplement: Supplementary file 6 [file gcb0017-1251-SD6.pdf]

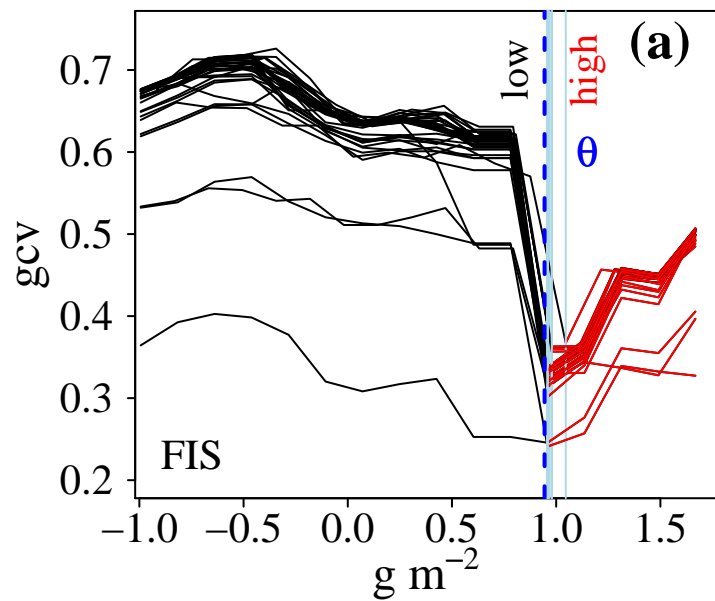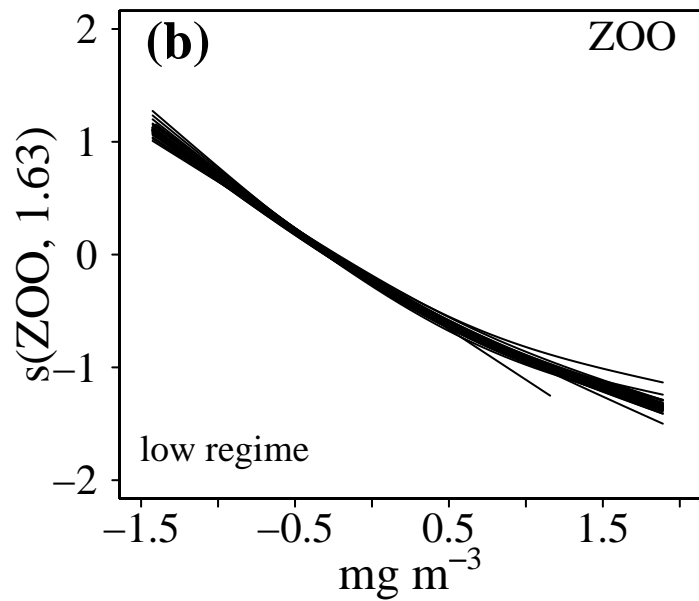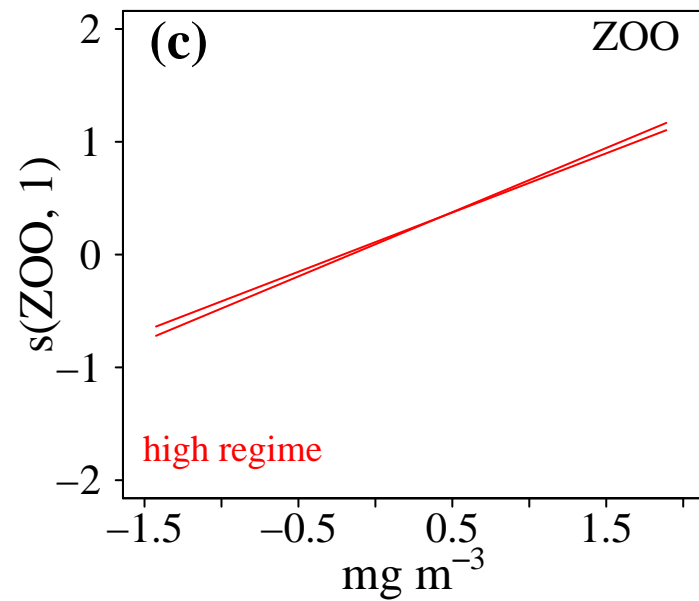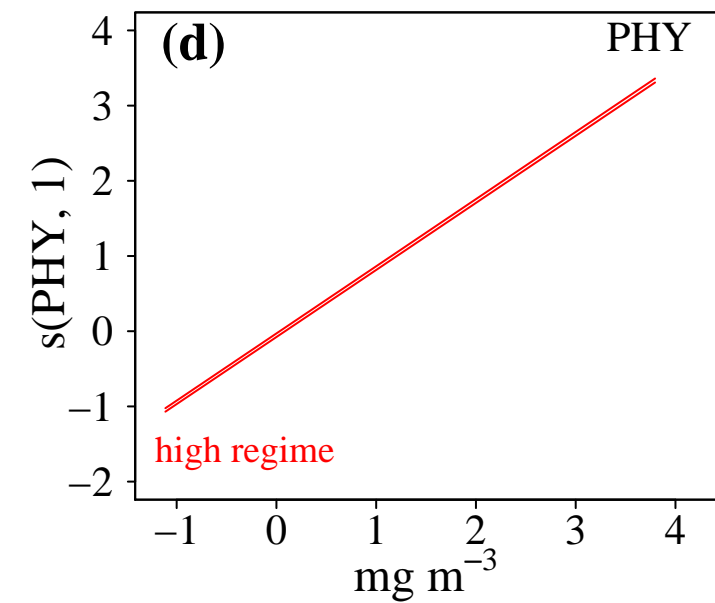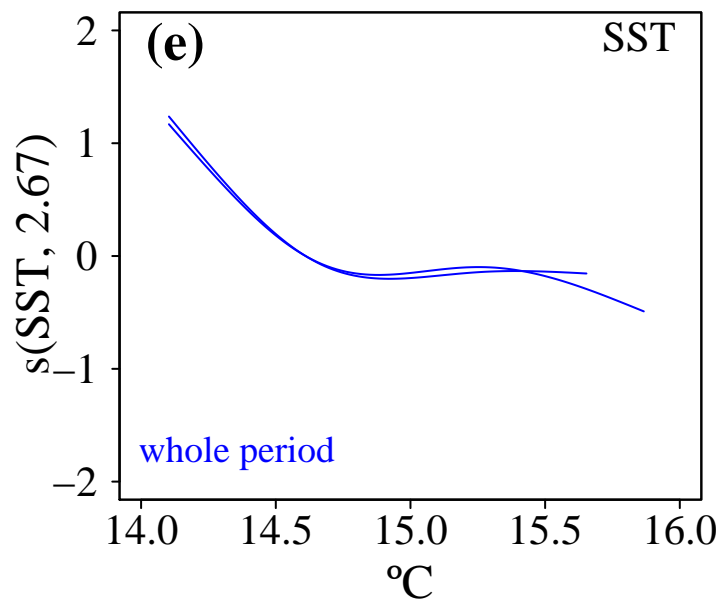

Supplement: Supplementary file 7 [file gcb0017-1251-SD7.pdf]

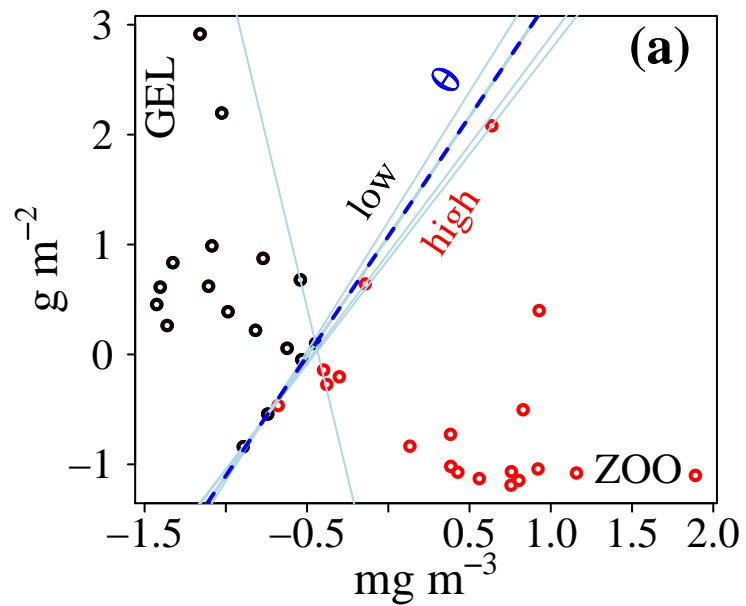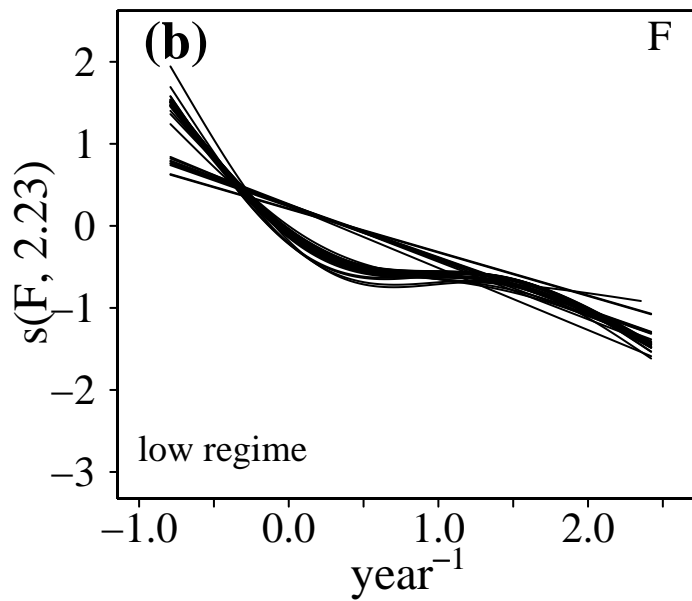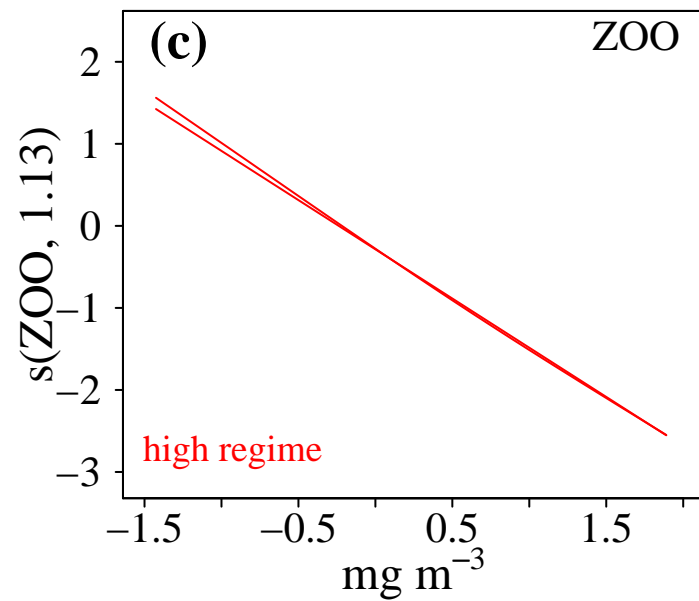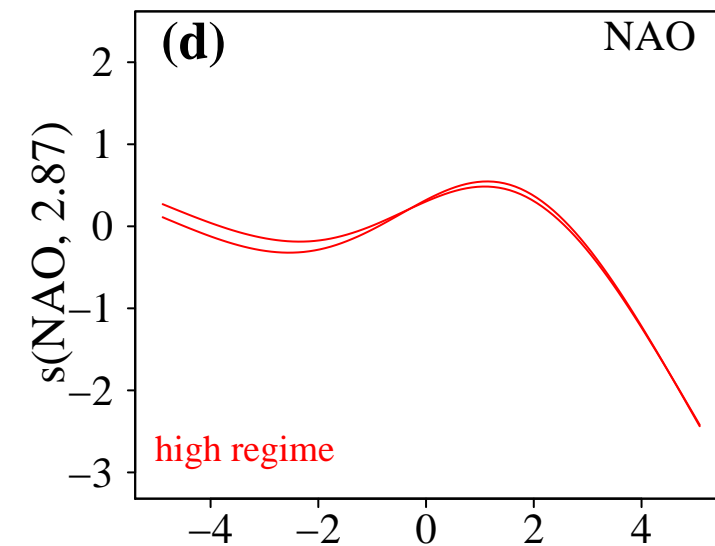

Supplement: Supplementary file 8 [file gcb0017-1251-SD8.pdf]

low P regimes

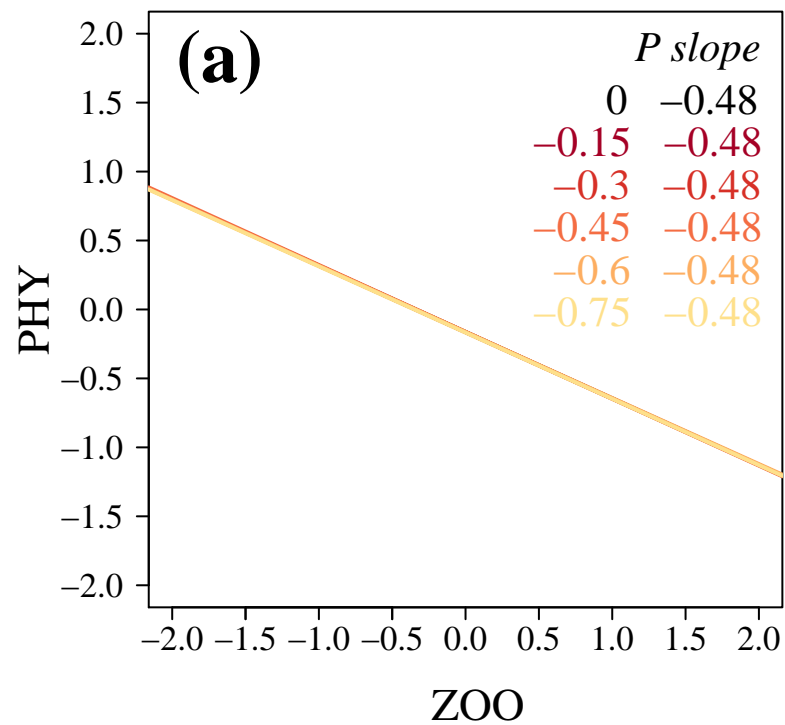

high P regimes

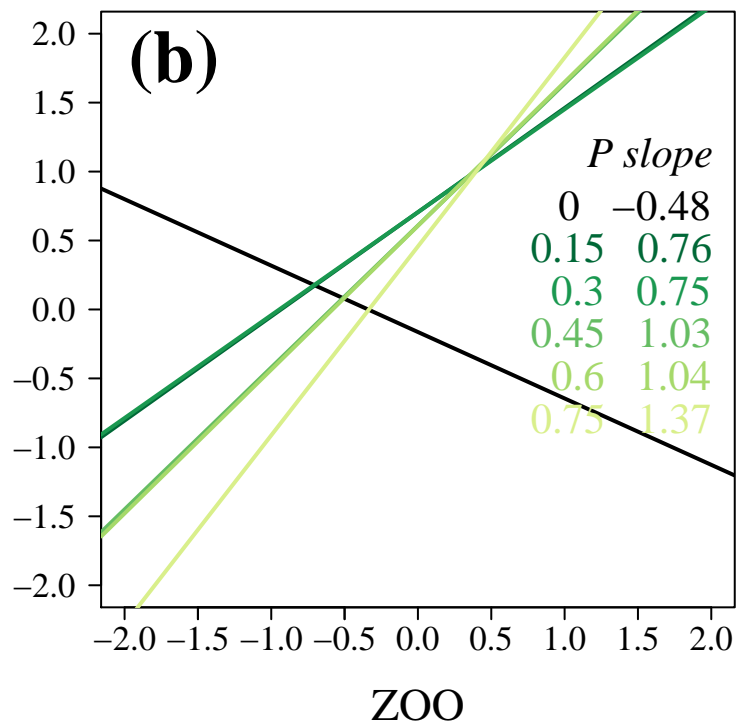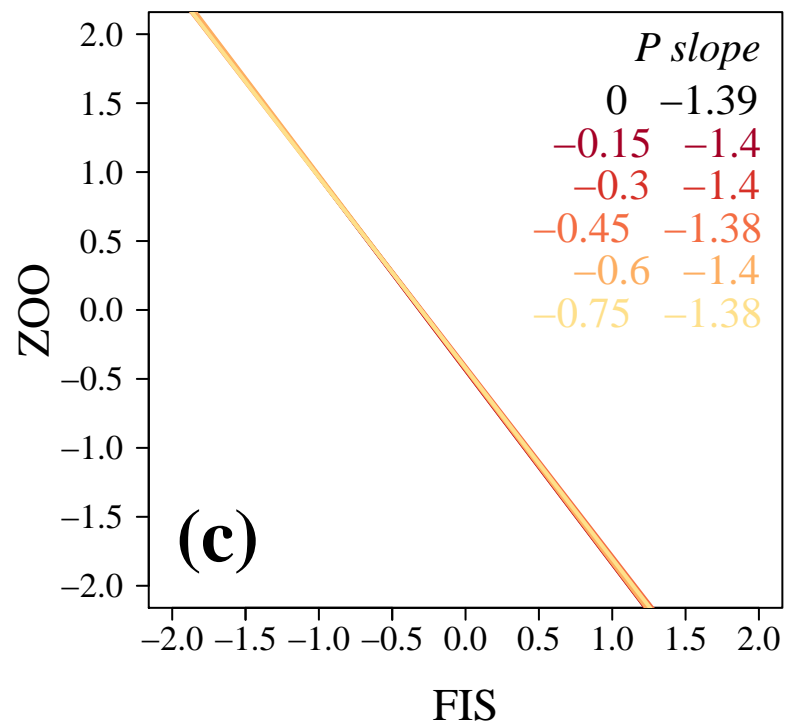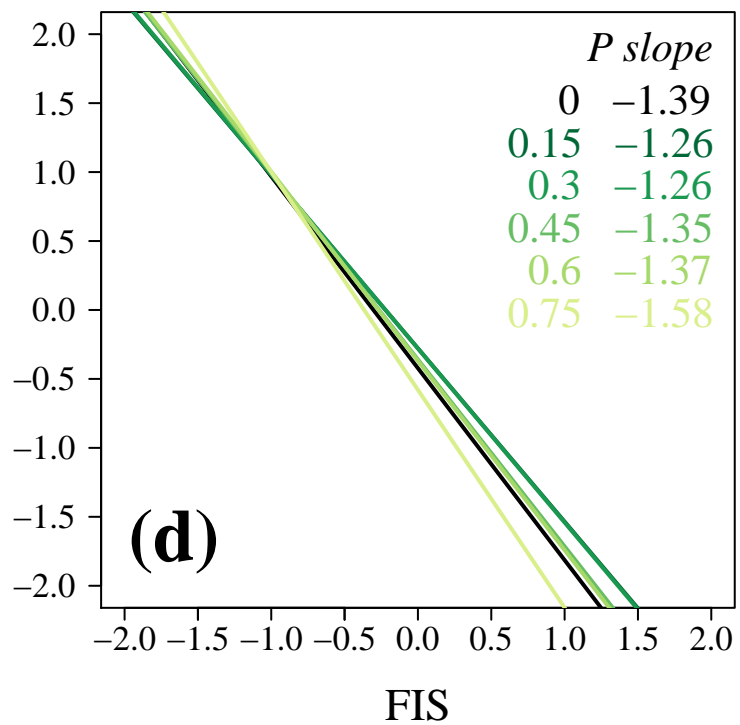

Supplement: Supplementary file 9 [file gcb0017-1251-SD9.pdf]

low P regimes

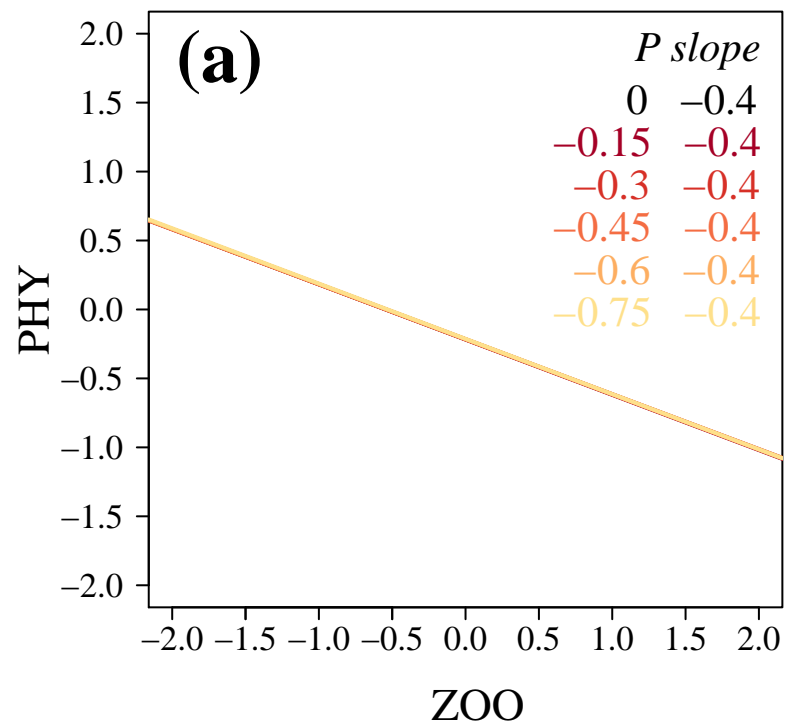

high P regimes

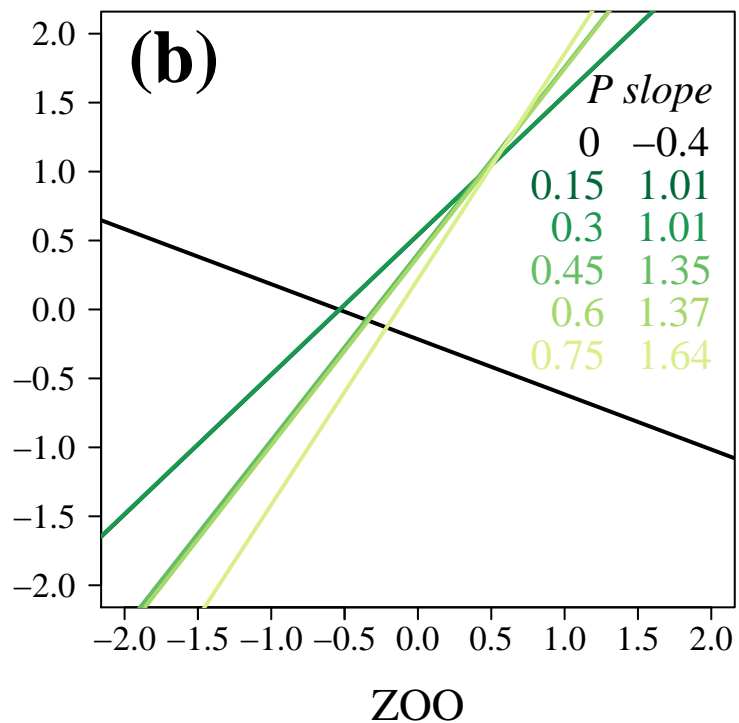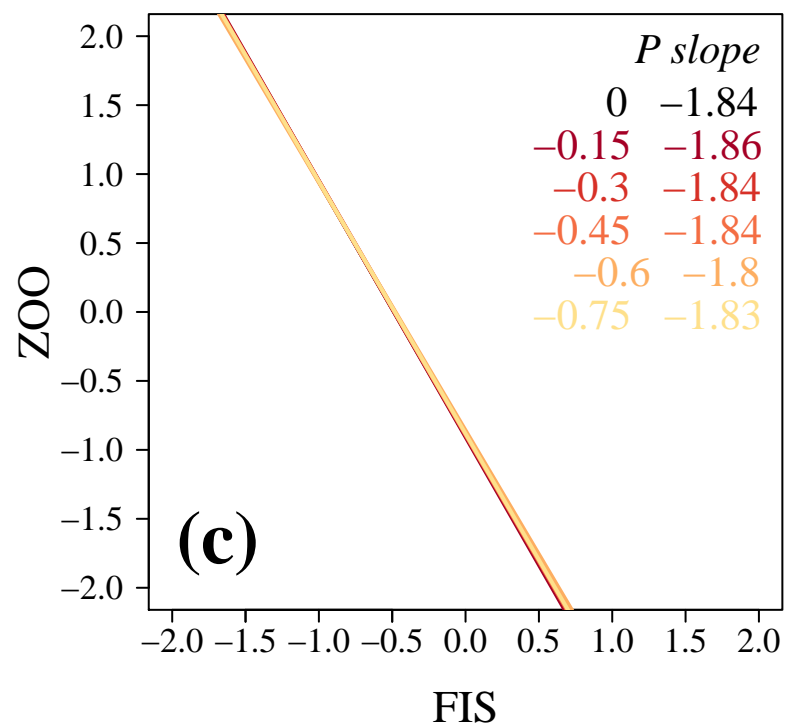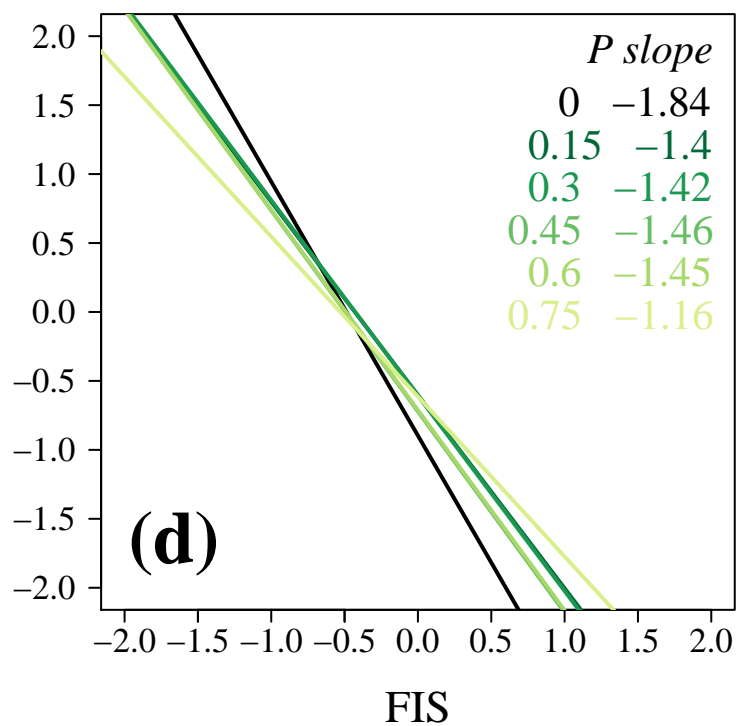

Supplement: Supplementary file 10 [file gcb0017-1251-SD10.pdf]

low FIS regimes

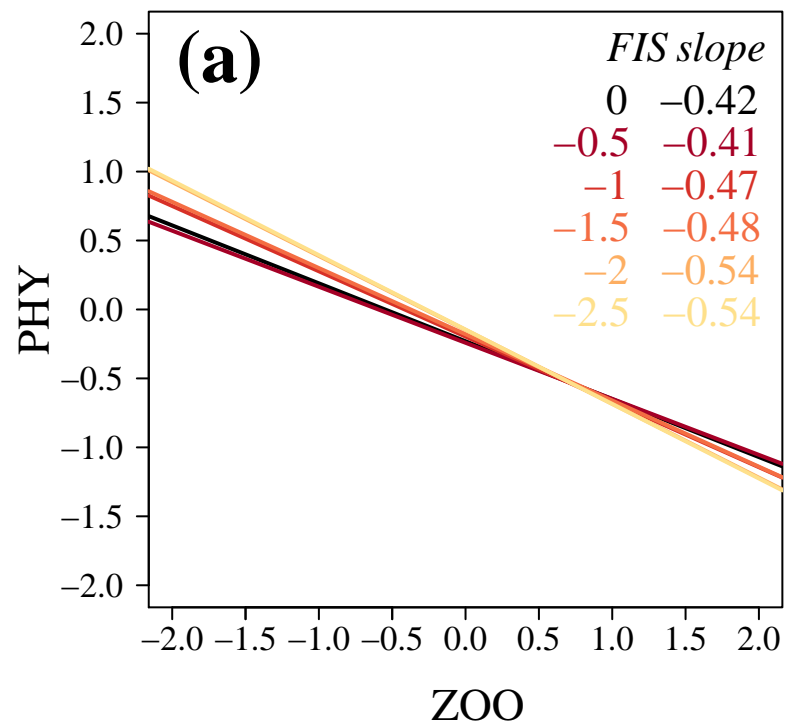

high FIS regimes

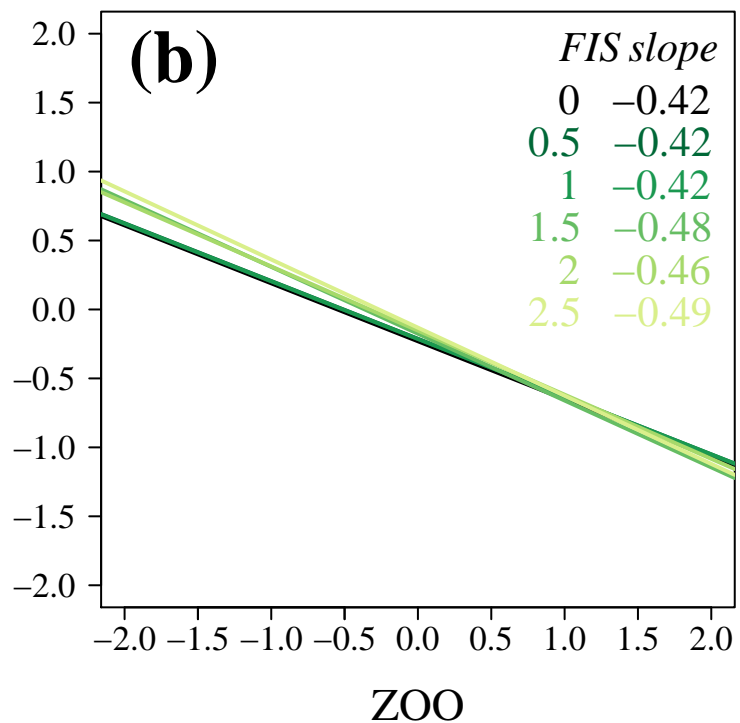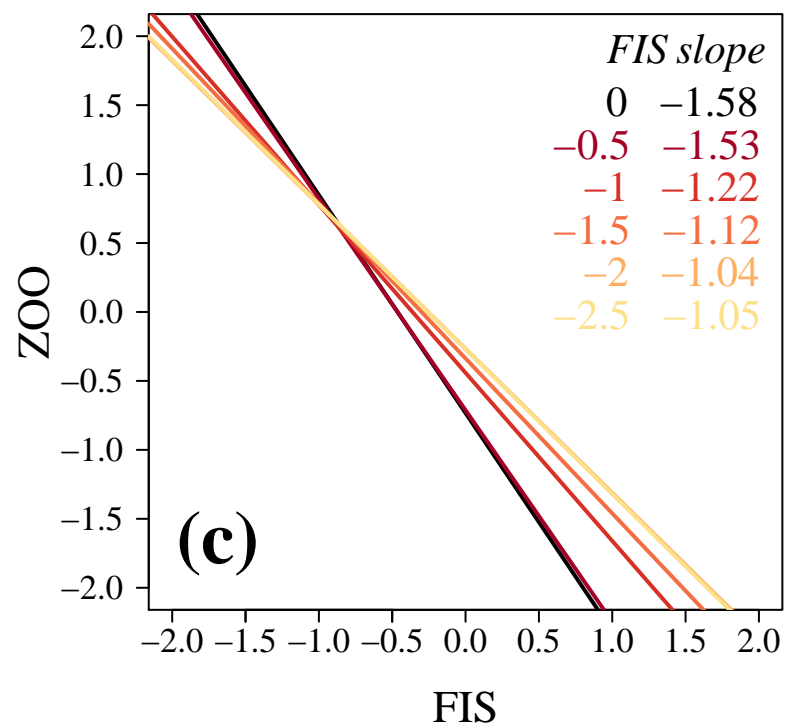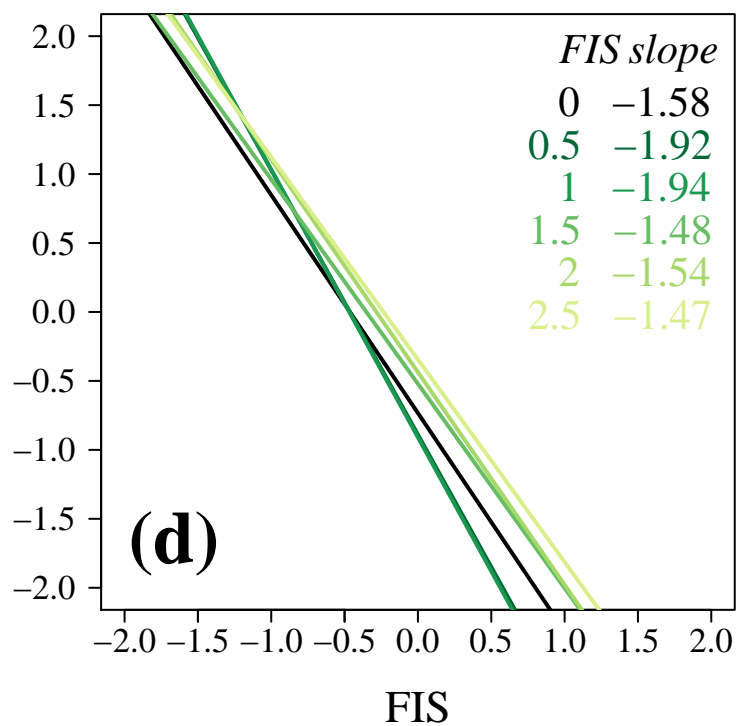

Supplement: Supplementary file 11 [file gcb0017-1251-SD11.pdf]

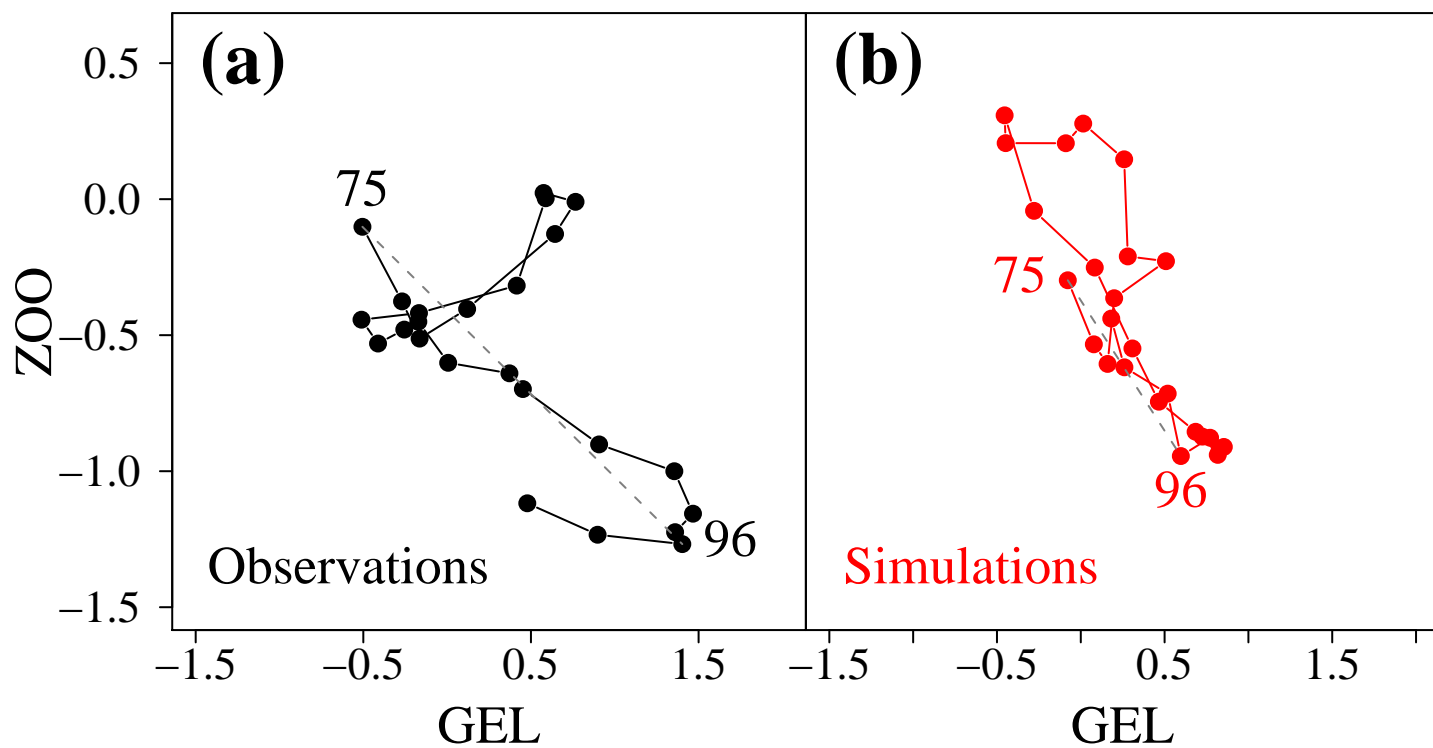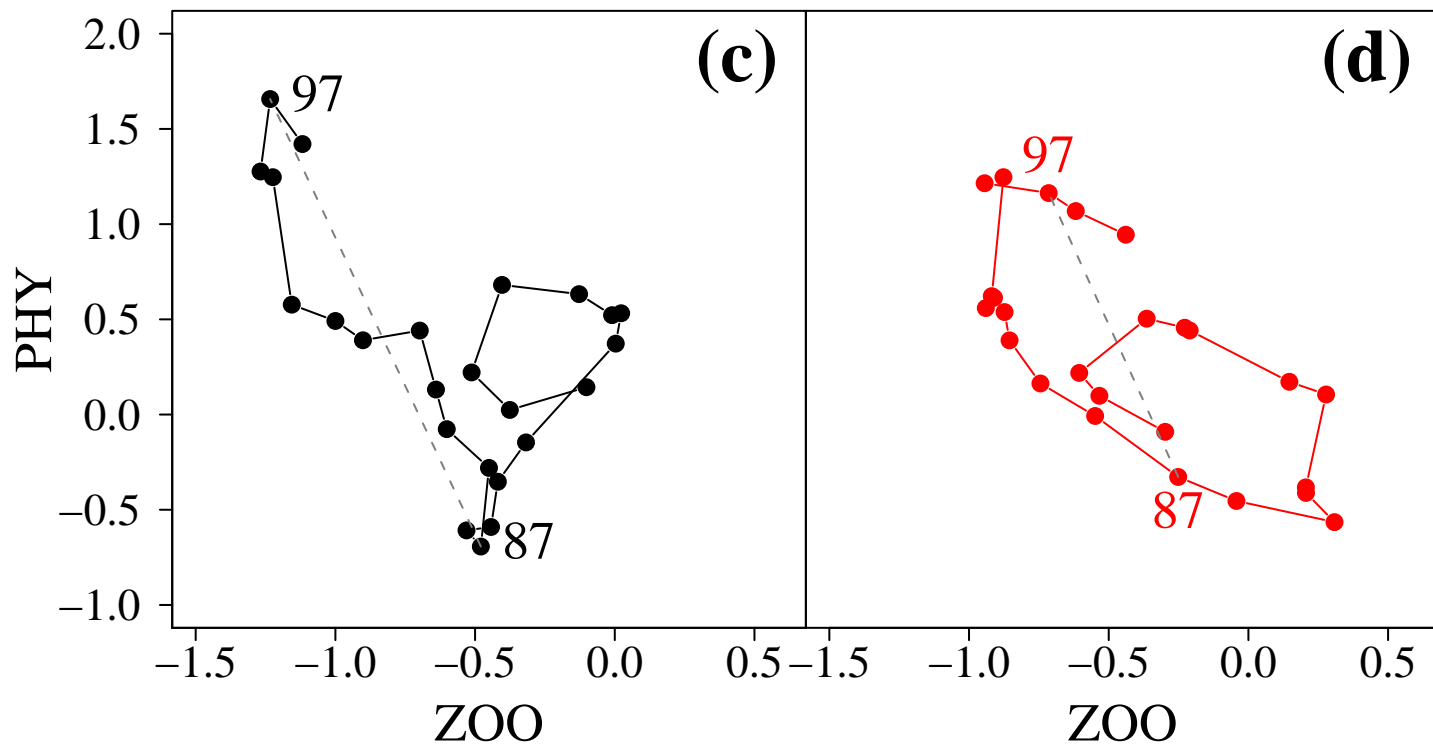

Supplement: Supplementary file 12 [file gcb0017-1251-SD12.pdf]

low P regimes

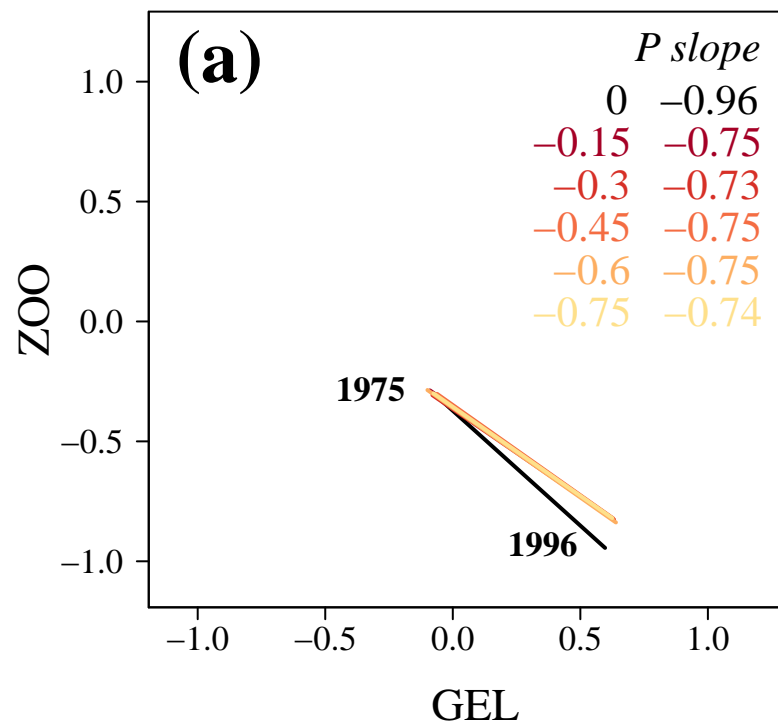

high P regimes

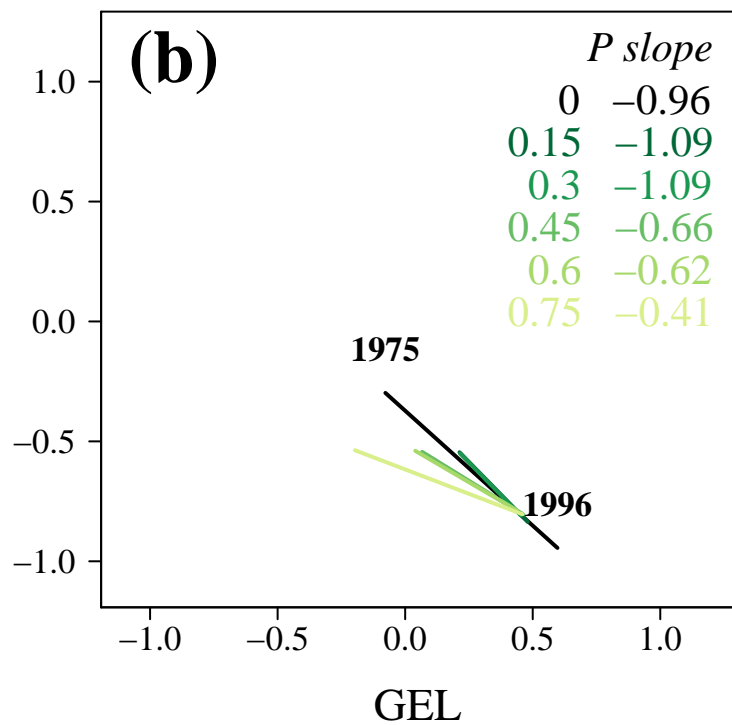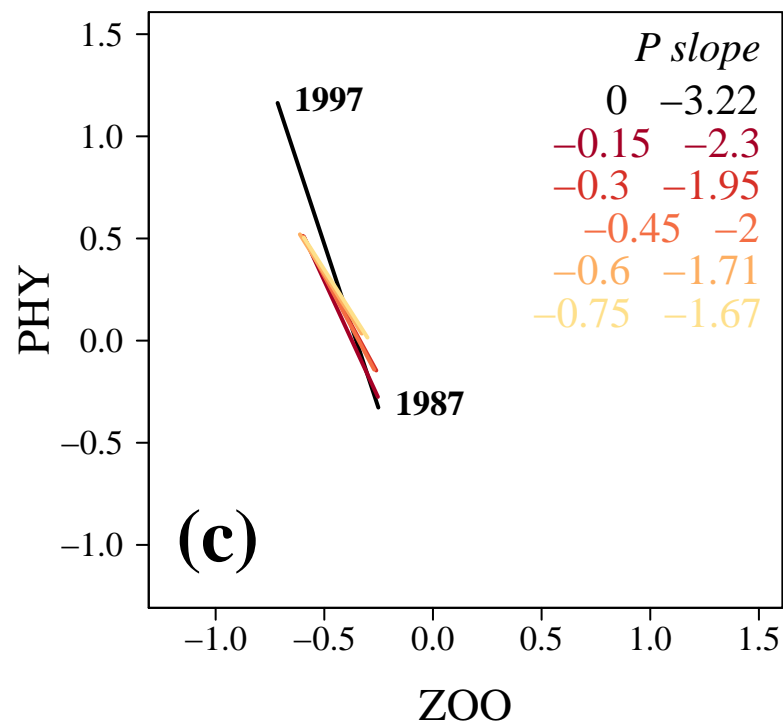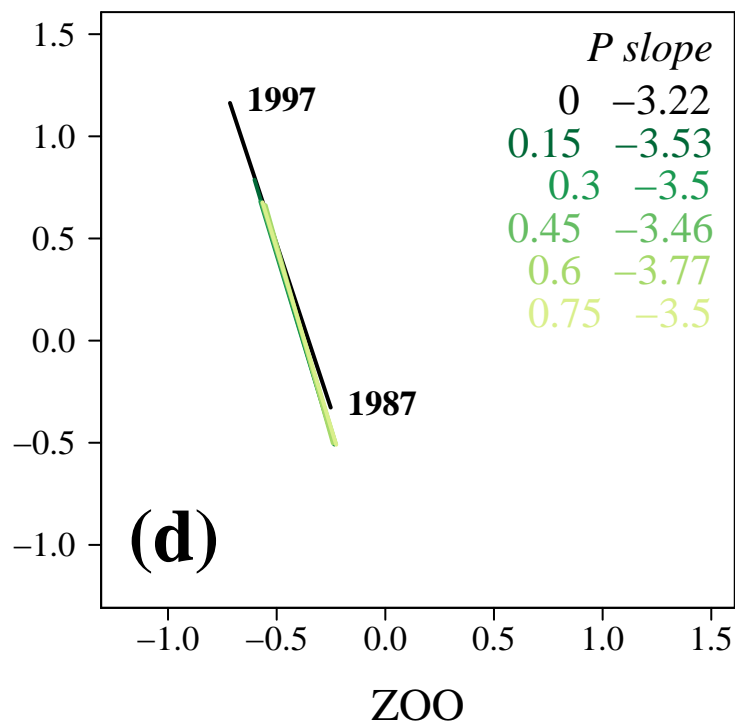

Supplement: Supplementary file 13 [file gcb0017-1251-SD13.pdf]

# low FIS regimes

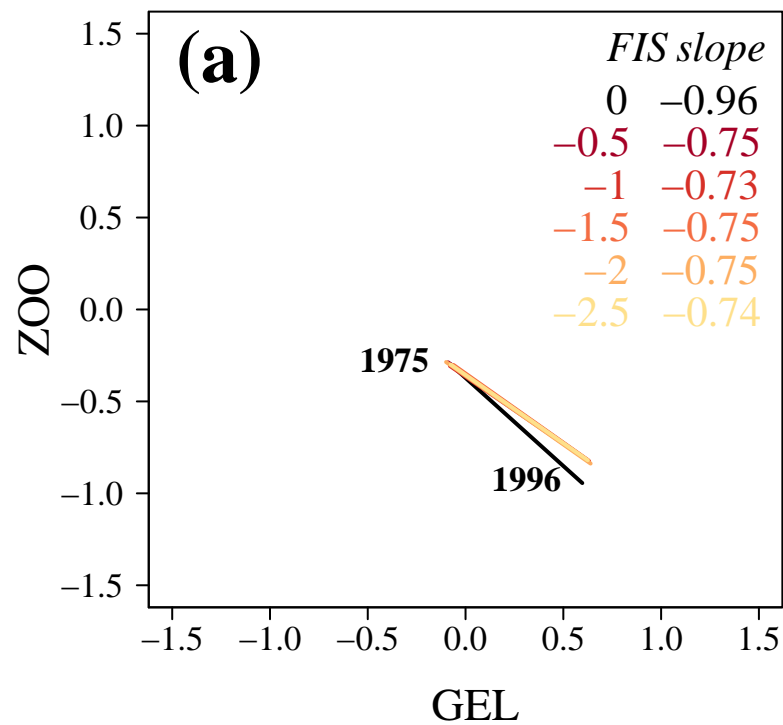

# high FIS regimes

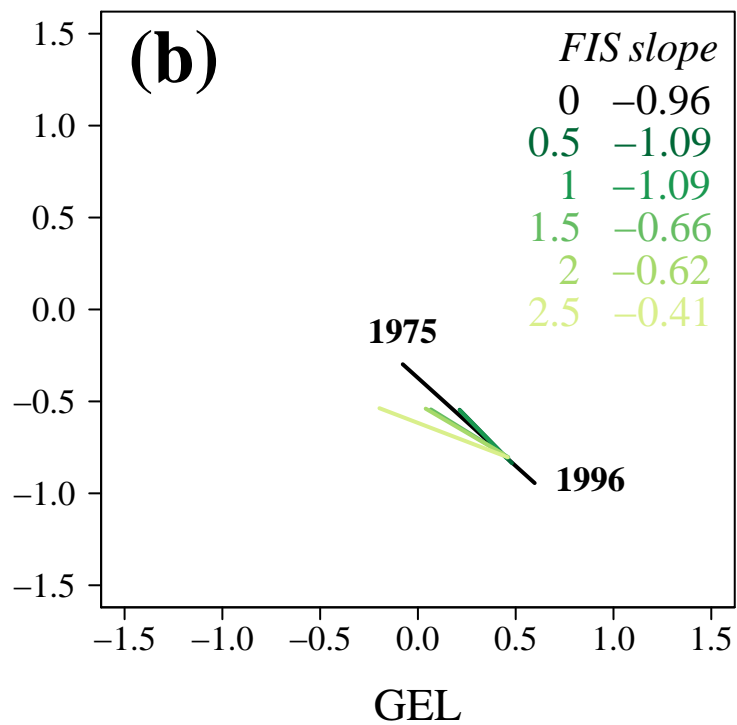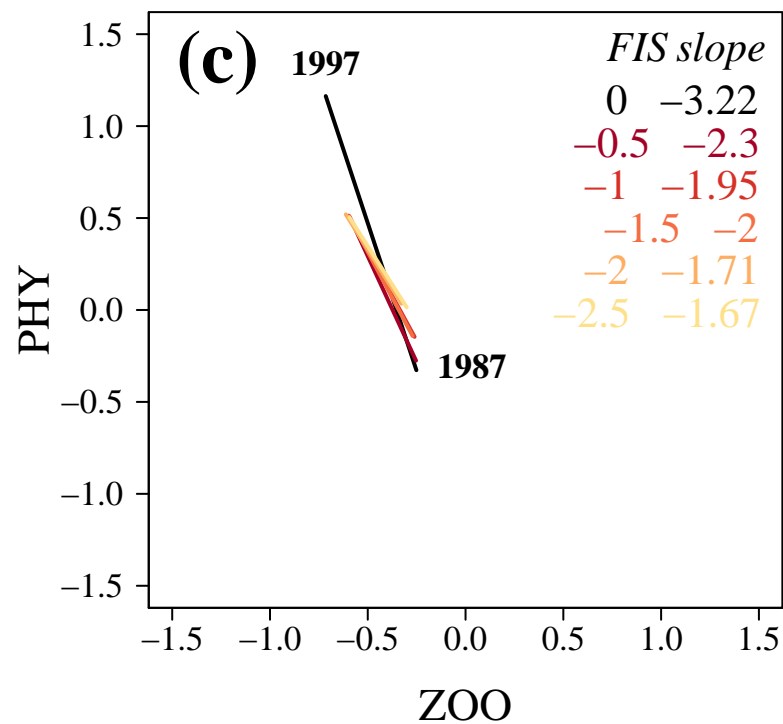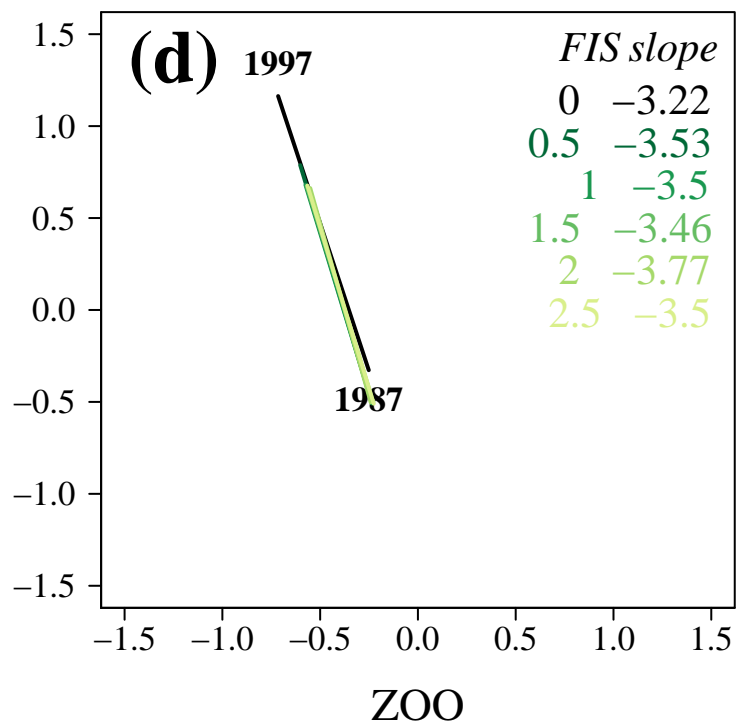

Supplement: Supplementary file 14 [file gcb0017-1251-SD14.pdf]
