# Supplementary material for: Overfishing of top predators eroded the resilience of the Black Sea system regardless of the climate and anthropogenic conditions
Source: Glob Chang Biol. 2011 Mar;17(3):1251–65. doi: 10.1111/j.1365-2486.2010.02331.x (PMC3597262; doi:10.1111/j.1365-2486.2010.02331.x)
Supplement: Supplementary file 15 [file gcb0017-1251-SD15.doc]

SUPPORTING INFORMATION

**Overfishing of top predators eroded the resilience of the Black Sea system regardless the climate and anthropogenic conditions**

M Llope, GM Daskalov, TA Rouyer, V Mihneva, K-S Chan, AN Grishin, & NC Stenseth

**Supporting text**

**Predatory fish**

The predatory fish biomass, as top trophic level, was not easy to model due to the lack of accurate estimates of fishing mortality. Two alternative models were selected, each of them including only one explanatory variable: planktivorous fish or zooplankton. In the model including planktivorous fish, this had a negative effect indicating strong top-down control. The one selected here (see Table S2), better in terms of R^2^ than that including planktivorous fish, showed a positive linear effect of zooplankton on predatory fish (Fig. S2a). These results reflect a strong cascade effect.

The predatory fish biomass (PRE) was used as a covariate but not simulated due to its low explanatory power.

**Gelatinous plankton**

In this model, the presence of two outliers corresponding to the second peak in the jellyfish biomass (being underestimated as compared to the rest of the series, see Fig. 1d) invalidates the normality and homoscedasticity assumptions for the residuals (Fig. S1c). To test the effect of these two outliers on the fitted model, we refitted the model by including two dummy variables accounting for the outliers. Overall, the model fit improved (R^2^ = 0.91) and it could now reproduce the second peak (Fig. S3i). The reported regression relationships remained significant (Table S3), even though the functional form changed slightly (Fig. S3b-e).

Since the main results from the previous model (Table 1 and Fig. 2) were not affected by these outliers we opted for keeping it so as not to further complicate the model fitting. Regarding the simulation experiments (scenarios), since we simulate by drawing from the residuals with replacement and randomly, it effectively accounts for the outliers by using a non-normal error distribution.

**Sensitivity Analysis**

To evaluate the robustness of the selected models (eqs. 1-4) we carried out a sensitivity analysis (cross validation). The cross validation consisted of three steps: (a) one data case is set aside for validation, (b) the model is fitted to the remaining (n-1) data cases, and (c) the ratio of the squared prediction error for that observation to the estimated noise variance of the latter model is computed (the ratio avoids scale issues). The average predictive ratio across all data cases (n=36) should be close to 1 if the model is correctly specified.

The averaged predictive ratio was 1.17 for phytoplankton, 1.76 for zooplankton, 1.55 for jellyfish, and 2.15 for fish. This suggests a higher sensitivity of the fish model (eq. 4), partly owing to the two-dimensional threshold structure. The out-of-sample predictions vs. observations are shown in Fig S4.

Furthermore, the 36 subset models for each of the four trophic levels were inspected and compared with the corresponding selected model. Figs. S5-S8 show the partial effects and threshold estimation superimposed for each of the 36 individual models. The fish model showed the highest variation including changes in the shape of partial effects (Fig. S8b), while these partial effects and the threshold for the rest of the groups proved to be insensitive and similar to those of the corresponding selected models.

**phase space approach**

*The 1970s’ shift.*

In the 1970s, the zoo/phytoplankton phase space plot showed an almost linear evolution (Fig. 5a-b); from a situation of low phytoplankton/high zooplankton in the late 1960s to high phytoplankton/low zooplankton at the end of the 1970s. This trajectory could be well represented by the slope of the regression line that joins all the points. The same applies to the zooplankton/fish plot (Fig. 5c-d). In order to illustrate these trajectories in a straight forward way –and by doing so be able to easily detect under which conditions (i.e., scenarios) there were significant trophic changes (i.e., shifts)– we chose to display the slope of this trajectories as a simplification of the observed phase space trajectories. Figs. S9 and S11 show a complete picture of all the phosphorus and fish scenarios tried respectively.

The simulated planktivorous fish biomasses for the 1970s captured the observed increasing trend but the levels of biomass were clearly underestimated (Fig. 4d). Although the phase space plots are based on the relative abundance of the trophic levels (not the absolute values) and the shift was detected for the trajectory of the two first trophic levels (not fish), the poor performance of the fish simulation required further analysis. To ensure the robustness of the result, the simulations for the different phosphorus scenarios were re-run, this time using the observations for fish (not the simulations). The results were basically the same (Fig. S10).

For the fish scenarios (Fig. S11), the biomass of planktivorous fish was not simulated but either decreased or increased based on the observations. This approach avoids any problem arising from the poor simulation performance.

*The 1990s’ shift.*

For the second major shift, occurred from the 1970s to the early 1990s, the situation is more complicated since it also includes the less significant reverse shifts of the 1980s and after 2000. This made the trajectories show loops and therefore they cannot be straightforward illustrated by linear trends. We chose to follow the evolution of the major axis (i.e., the line joining the two most distant points): 1975-1996 for jellyfish and zooplankton and 1987-1997 for zooplankton and phytoplankton (Fig. S12). The slopes of those lines were tracked in Figs. S13 and S14. No drastic changes were found.

**Supporting tables**

**Table S1** gCVs. Genuine Cross-validation scores (gCV) for the non-additive GAM model (Threshold GAM) and the fully additive equivalent for all the trophic levels.

| Model | gCV | |
| --- | --- | --- |
|  | Non additive model | Additive model |
| Phytoplankton | 0.5826 | 0.9989 |
| Zooplankton | 0.4814 | 0.7017 |
| Gelatinous plankton | 0.5891 | 0.9507 |
| Planktivorous fish | 0.8265 | 0.8812 |
|  |  |  |

**Table S2** Predatory fish (PRE) models results. Intercept, estimated degrees of freedom (edf) and significance (p-value) of the covariate, and R-squared (R^2^) for the predatory fish model (see Fig. S2). It also includes a dummy variable accounting for the effect of an outlier (o1).

|  |  |  |  |  |
| --- | --- | --- | --- | --- |
|  | PRE | | |  |
|  |  | estimate | p-value |  |
|  | intercept | -0.33 | <0.001 |  |
|  | o1 | 2.70 | <0.001 |  |
|  | threshold | none |  |  |
| regime | covariate | edf | p-value |  |
| - | ZOO | 1.00 | <0.001 |  |
|  | R^2^ (adj) = 0.618 | | |  |
|  |  |  |  |  |

**Table S3** GEL models results – accounting for outliers. Intercept, estimated degrees of freedom (edf) and significance (p-value) of the various effects, and R-squared (R^2^) for the gelatinous plankton model when including two dummy variables (o1 and o2) targeting the two outliers observed in the residuals (Fig. S1c). This model summary corresponds to Fig. S3.

|  | GEL | | |  |
| --- | --- | --- | --- | --- |
|  |  | estimate | p-value |  |
|  | intercept | -0.16 | 0.0125 |  |
|  | o1 | 2.01 | <0.001 |  |
|  | o2 | 1.84 | <0.001 |  |
|  | threshold (θ) | 1.12 |  |  |
| regime | covariate | edf | p-value |  |
| FIS≤ θ | ZOO | 2.35 | <0.001 |  |
| FIS> θ | ZOO | 2.41 | 0.003 |  |
| FIS> θ | PHY | 3.00 | <0.001 |  |
| - | SST | 2.09 | 0.005 |  |
|  | R^2^ (adj) = 0.906 | | |  |

**Supporting figure legends**

**Figure S1** Thresholds and residuals. Regime assignment and residuals for each of the four individual models: phytoplankton (a), zooplankton (b), gelatinous plankton (c), and planktivorous fish (d). The first column (a-c) shows the temporal evolution of the threshold variables (phosphorus, fish, and fish, respectively) and their regime allocation: those points above the threshold (represented by the blue line, θ) are shown in red (high regime) while those below appear in black (low regime). The bivariate threshold of the fish model (d) is explained in Fig. 2 (see legend there for details). The following four columns show the inspection of residuals for the assumptions of independence (autocorrelation function), normality (Q-Q plot), and homoscedasticity (residuals vs. time and residuals vs. fitted values). Apart from visual inspection, Shapiro (normality) and Breusch-Pagan (homoscedasticity) tests were performed (values not shown). As commented earlier (see text), only jellyfish residuals violated the latter assumptions.

**Figure S2** Predatory fish model. Plots showing the effect of zooplankton on predatory fish (a), observations vs. predictions (b), and residual statistics: autocorrelation (c) and normality (d).

**Figure S3** Alternative gelatinous model partial effects and residuals. Plots showing the estimation of the threshold value (a), all the partial effects (b-e), regime assignment to threshold variable (f), residual statistics (g-h), and predictive performance (i) for the gelatinous plankton model when accounting for the two outliers detected in the residuals.

**Figure S4** Observations (black) and out-of-sample predictions (purple) of the biomasses of phytoplankton (a), zooplankton (b), gelatinous plankton (c), and planktivorous fish (d).

**Figure S5** Threshold estimation (a) and partial plots (b-d) for the phytoplankton cross validation models. Each plot consists of the overlaid results from the 36 subset models. The numbers in parentheses on the y-axis indicate the averaged estimated degrees of freedom. See also Fig. 2 legend for further details.

**Figure S6** Threshold estimation (a) and partial plots (b-e) for the zooplankton cross validation models calculated as for PHY (see legend in Fig. S5).

**Figure S7** Threshold estimation (a) and partial plots (b-e) for the jellyfish cross validation models calculated as for PHY (see legend in Fig. S5).

**Figure S8** Threshold estimation (a) and partial plots (b-d) for the fish cross validation models calculated as for PHY (see legend in Fig. S5).

**Figure S9** 1970s phase space plots for all the phosphorus scenarios. Illustration of the phase space trajectories for zooplankton and phytoplankton (a-b), and planktivorous fish and zooplankton (c-d) for all the phosphorus regimes during the 1970s major shift. The lower phosphorus regimes are shown to the left (scale of reds) and the higher to the right (greens). The legend shows the value of the slopes to the corresponding percentage of the mean increase or decrease.

**Figure S10** 1970s phase space plots for all the phosphorus scenarios (fish observations). Same as Fig. S9 but using observations for fish biomass, not simulations.

**Figure S11** 1970s phase space plots for all the fish scenarios. As Fig. S9 but for fish regimes, lower abundance to the left (reds) and higher to the right (greens).

**Figure S12** Observations vs. simulations phase space plots. Phase space plots of consumer (driver) against resource (response) for the observations (left column, in black) and simulations (right column, in red) during the 90s regime shift. Standardized data from Fig. 4 are used. Numbers on the plots are years. Dashed lines are the slopes of the linear regression lines joining all the points.

**Figure S13** 1990s phase space plots for all the phosphorus scenarios. Illustration of the phase space trajectories for gelatinous plankton and zooplankton (a-b), and zooplankton and phytoplankton (c-d) for all the phosphorus regimes during the 1990s second major shift. The lower phosphorus regimes are shown to the left (reds) and the higher to the right (greens). The legend shows the value of the slopes to the corresponding percentage of the mean increase or decrease.

**Figure S14** 1990s phase space plots for all the fish scenarios. As Fig. S13 but for fish regimes, lower abundance to the left (reds) and higher to the right (greens).
